# Angle-dependent X-ray absorption correction in small- and wide-angle X-ray scattering: accounting for constrained scattering volume, beam profiles and capillary wall attenuation

**DOI:** 10.1107/S1600576726006837

**Published:** 2026-08-06

**Authors:** Jure Kovač, Andrej Jamnik, István Szilágyi, Matija Tomšič

**Affiliations:** a Faculty of Chemistry and Chemical Technology University of Ljubljana Večna pot 113 Ljubljana1000 Slovenia; b MTA-SZTE Momentum Biocolloids Research Group, Department of Physical Chemistry and Materials Science, Interdisciplinary Research Center University of Szeged SzegedH-6720 Hungary; Argonne National Laboratory, USA

**Keywords:** transmission correction coefficients, X-ray absorption correction, fluorinated liquids, Kratky cameras, absolute scaling, wide-angle X-ray scattering, WAXS, small-angle X-ray scattering, SAXS, perfluorinated alkyl substances, PFAS

## Abstract

A GPU-accelerated numerical method has been developed to calculate angle-dependent transmission factors *A*(2θ) for small- and wide-angle X-ray scattering (SWAXS) data collected with a Kratky-type camera using a line-collimated beam and a horizontal cylindrical capillary with a vertically oriented detector. The procedure extends path-length-based absorption correction to the Kratky-type geometry by averaging Beer–Lambert attenuation over the constrained illuminated scattering volume and by including experimentally measured primary beam profiles and capillary-wall absorption. Sensitivity analysis shows that the vertical illumination geometry dominates the calculated angle-dependent transmission factor, whereas the horizontal beam-width effect is negligible. Using measured beam profiles makes the correction instrument specific and avoids introducing an effective uniform beam height as an approximation. For strongly absorbing nona­fluoro-*tert*-butanol, the conventional scalar primary beam transmission correction fails after subtraction of empty-capillary scattering, while the numerical correction enables physically meaningful absolute-scale data reduction. Comparisons between independently calculated theoretical SWAXS intensities and the two reduced experimental datasets obtained sequentially using two different detectors in the same experiment on the same Kratky-type SWAXS camera provide a direct experimental cross check for the data-reduction workflow based on calculated transmission factors. They indicate that the observed limitation is associated with transmission measurement of a spectrally modified primary beam, rather than with the Kratky-type geometry itself. Importantly, the results show that the limitations of conventional scalar transmission corrections are not confined to extreme cases but may also affect routine water-based absolute-scale SWAXS calibration. A simple Beer–Lambert-type numerical scalar correction can still provide a useful qualitative approximation, whereas reliable quantitative analysis requires the full angle-dependent *A*(2θ) correction.

## Abstract 2

We present an improved numerical angle-dependent absorption correction procedure for line-collimated small- and wide-angle X-ray scattering measurements in thin-walled quartz capillaries. The procedure accounts for the constrained illuminated volume, measured primary beam profiles and capillary-wall absorption, enabling physically consistent data reduction and reliable absolute scaling when conventional experimental scalar transmission corrections become unreliable.

## 1. Introduction

When performing X-ray scattering measurements on a series of samples, reliable absorption correction is essential for basic data reduction, because subsequent background subtraction and data interpretation critically depend on it. We focus our attention on small- and wide-angle X-ray scattering (SWAXS, including both SAXS and WAXS) measurements of liquid and soft-matter samples in cylindrical quartz capillaries performed using line-collimated Kratky-type cameras (Fritz & Bergmann, 2006 ▸), which remain established laboratory instruments in colloid and soft-matter research. The broader motivation for this study is summarized in Appendix 1 of the supporting information (Cerar *et al.*, 2020 ▸; Tomšič, Jamnik *et al.*, 2007 ▸; Lajovic *et al.*, 2010 ▸; Cerar *et al.*, 2021 ▸; Cerar *et al.*, 2019 ▸; Tomšič *et al.*, 2019 ▸; Tomšič *et al.*, 2018 ▸; Cerar *et al.*, 2017 ▸; Lajovic *et al.*, 2012 ▸; Lajovic *et al.*, 2009 ▸; Tomšič, Vlček *et al.*, 2007 ▸). Note that the supporting information is a substantial document that forms an integral part of this paper, providing extensive details on all aspects of the discussion, and is intended to be read alongside the main text. As such, frequent references to it are made in the following.

In a conventional SAXS/SWAXS workflow, each raw curve *I*_*j*,raw_(2θ) is first corrected using an experimental scalar transmission factor *T*_*j*_,
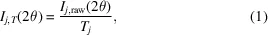
where *I*_*j*,*T*_(2θ) represents the absorption-corrected curve, and *j* = s, sol, w or ecap denotes the sample, solvent, water or empty-capillary measurement, respectively. In Kratky-type cameras with a semi-transparent beamstop, the factor *T*_*j*_ is determined from the primary beam transmission measured for the corresponding measurement, *i.e.* derived from the measured primary beam intensity at zero scattering angle, *I*_*j*,raw_(0). The corresponding background scattering (solvent or empty capillary) is then subtracted, yielding the background-corrected SWAXS data for the sample *I*_s,bc_(2θ) and water *I*_w,bc_(2θ),
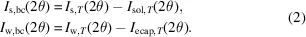
The latter expression is important if the SWAXS data need to be put on an absolute scale, as the absolute scaling factor *F*_*T*_ can be calculated using water as a secondary standard (Orthaber *et al.*, 2000 ▸) as
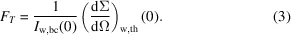
Here, (dΣ/dΩ)_w,th_(0) is the known theoretical scattering cross section of water at zero scattering angle (1.632 × 10^−2^ cm^−1^ at 293 K) (Orthaber *et al.*, 2000 ▸) and *I*_w,bc_(0) is obtained by extrapolating the experimental *I*_w,bc_(2θ) data to the zero scattering angle. Absolute scaling is then performed as
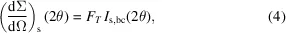
where (dΣ/dΩ)_s_(2θ) is the absolute-scale differential scattering cross section of the sample. For interested readers, additional comments are provided in Appendix 2 of the supporting information (Orthaber *et al.*, 2000 ▸; Pauw, 2013 ▸; Bergmann *et al.*, 2000 ▸).

This conventional procedure usually works well in SAXS/SWAXS on Kratky-type cameras when the attenuation of all measurements in a series is similar, for example for dilute samples and their corresponding solvent. However, it may become unreliable when the X-ray absorption differs substantially between the measured samples. Thus, the issue is not the conventional data-reduction workflow itself but rather the practical limitation of experimentally determining reliable scalar transmission factors *T*_*j*_. We attribute this limitation primarily to differences in the degree of primary beam radiation hardening between samples with substantially different attenuation properties, an effect further enhanced by the absorption of the semi-transparent beamstop in Kratky-type instruments. SAXS/SWAXS position-sensitive detectors, including modern ones, generally provide no or only limited photon energy discrimination for the detected beam, so the measured primary beam intensities can become strongly biased in such cases (Baur *et al.*, 2019 ▸; Hövelmann *et al.*, 2019 ▸). We discuss primary beam radiation hardening in more detail in Appendix 3 (Bergmann *et al.*, 2000 ▸; Oberta *et al.*, 2012 ▸; Kirschbaum *et al.*, 1997 ▸; Shimizu & Omote, 2008 ▸; Baur *et al.*, 2019 ▸; Brooks & Di Chiro, 1976 ▸; Van de Casteele *et al.*, 2002 ▸; Hövelmann *et al.*, 2019 ▸; Wu & Li, 2024 ▸; Polikarpov *et al.*, 2014 ▸; Chen *et al.*, 2018 ▸). Appendix 4 presents a practical example of the failure of the conventional procedure for the strongly absorbing liquid nona­fluoro-*tert*-butanol (NFTB). NFTB is therefore used as a representative test case in this study.

A way of overcoming such experimental problems is to resort to calculated transmission factors from numerical absorption correction procedures, which can also include more sophisticated treatments, *e.g.* angle-dependent self-absorption correction. Numerous numerical procedures have been developed and tested over many decades to correct scattering data for X-ray absorption. These methods place particular emphasis on sample geometry and the X-ray path through the material in different experimental setups, as reviewed in Appendix 5 (Claassen, 1930 ▸; Bradley, 1935 ▸; Bond, 1959 ▸; Dwiggins, 1975 ▸; 1972 ▸; Ritter *et al.*, 1951 ▸; Møller & Jensen, 1952 ▸; Schmitt & Ouladdiaf, 1998 ▸; Bowden & Ryan, 2010 ▸; Pauw, 2013 ▸; Pauw *et al.*, 2017 ▸; Bendert *et al.*, 2013 ▸; Van Hoesen *et al.*, 2019 ▸; Jeffries *et al.*, 2021 ▸; Sulyanov *et al.*, 2012 ▸). Here we focus on the procedure of Bowden & Ryan (2010 ▸), who treated absorption in cylindrical and annular specimens and their containers or supports within a powder diffraction framework based on cross-sectional path-length integration. This makes their work the closest reference point for the cylindrical capillary geometry relevant to line-collimated Kratky-type SAXS and SWAXS instruments.

The novelty of our work is the extension of this type of path-length-based correction towards the actual geometry of line-collimated SWAXS instruments using routinely available instrument-specific information, namely the experimentally measured non-uniform primary beam profiles. In this way, the finite constrained illuminated scattering volume is retained, but its contribution is weighted by the measured primary beam intensity distribution rather than being treated as uniformly illuminated. In addition, we do not treat the capillary wall only as an absorbing medium along the photon path but also consider its scattering contribution separately in the empty-capillary measurement. These elements are then incorporated into the usual data reduction sequence, in which the experimental scalar transmission factor is replaced by calculated angle-dependent transmission factors before background subtraction and absolute scaling.

The objective of the present work is therefore to formulate, implement and evaluate a numerical angle-dependent absorption correction procedure directly applicable to line-collimated SWAXS measurements of liquid samples in thin-walled cylindrical capillaries. The procedure is designed to address the limitations of scalar transmission-based data reduction and absolute scaling that we encountered in our SWAXS studies of strongly absorbing samples, as discussed in detail in Appendix 4. We evaluate the procedure by testing the numerical convergence of the calculated transmission factors and by analysing their sensitivity to beam geometry, measured beam profiles and sample attenuation in Appendix 8. We further apply it to experimental SWAXS datasets from chemically diverse systems in Appendix 10 (Pi *et al.*, 2009 ▸; Cerar *et al.*, 2021 ▸). These applications also provide an important model-based consistency check by comparing corrected experimental SWAXS data with independently calculated theoretical SWAXS intensities for the same systems, obtained using the ‘complemented system approach’ (Cerar *et al.*, 2020 ▸; Tomšič, Jamnik *et al.*, 2007 ▸; Lajovic *et al.*, 2010 ▸). These theoretical intensities are calculated from molecular simulation results and are not fitted, scaled or otherwise adjusted to match the experimental data, thereby representing a fully independent reference dataset within the limitations of the chosen molecular force field. In addition, measurements performed on the same sample in the same capillary and on the same Kratky-type camera, without changing the sample/capillary arrangement but with a Dectris Mythen 1K detector, are used as a direct experimental cross check of the proposed absorption correction workflow, as discussed in Appendix 11.

## 2. Formulation of a realistic SWAXS absorption correction model

**Figure 1:**
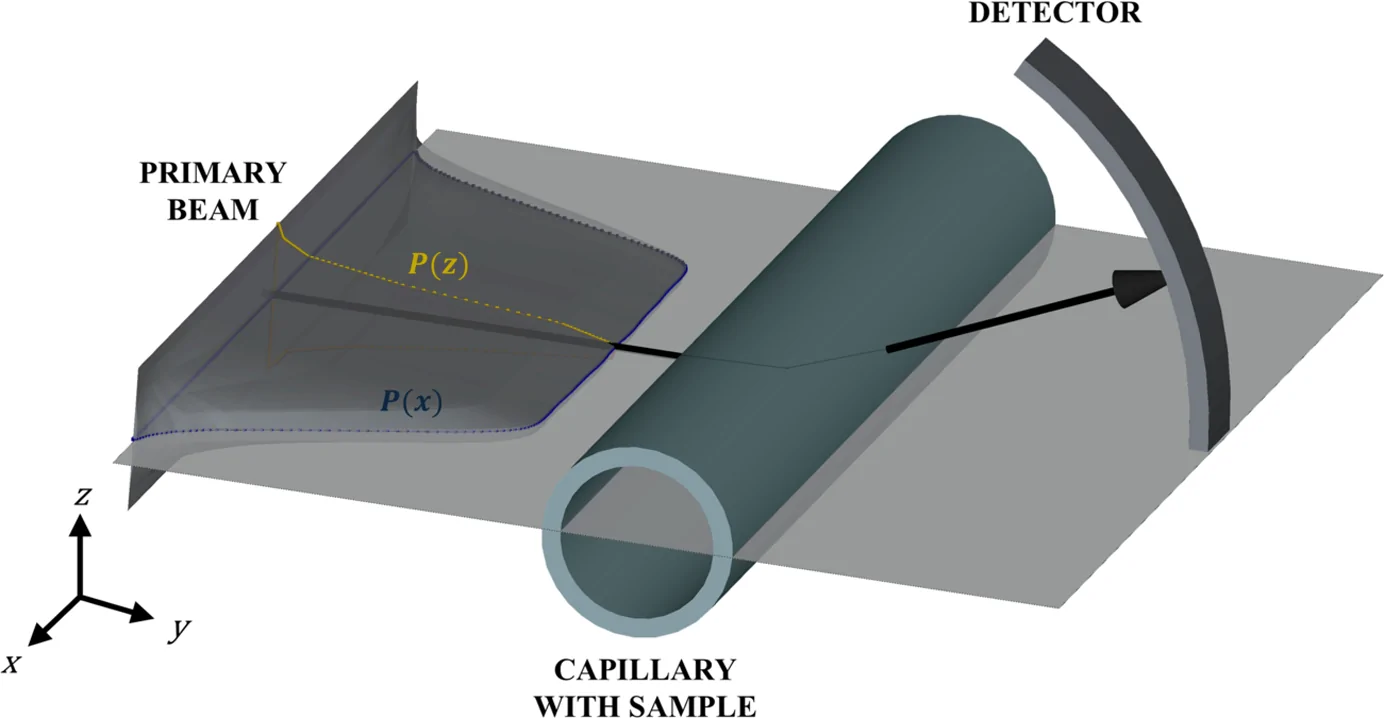
Schematic representation of the considered experimental geometry.

We focus on Kratky-type cameras and similar modern line-collimated SWAXS geometries (Glatter, 2018 ▸) in which a thin-walled horizontal quartz capillary is illuminated by a horizontally collimated primary beam passing through its central axis. In such instruments, the capillary is consistently aligned to ensure it is always centrally illuminated. The detector is positioned perpendicular to the capillary axis and oriented vertically, with the scattering signal recorded in the upward direction, as depicted schematically in Fig. 1 ▸. For this geometry, we extend the existing path-length-based absorption correction framework (Bowden & Ryan, 2010 ▸) by incorporating weighting functions based on experimentally determined primary beam profiles, constraining the scattering volume vertically by the beam’s ‘height’ and horizontally by its ‘width’ profile, both of them perpendicular to the beam propagation direction. Here, beam height and beam width denote the beam height in the vertical (*zy*) plane and the beam width in the horizontal (*xy*) plane, respectively. The introduced weighting functions provide a direct way to connect idealized path-length calculations with the actual illumination conditions in a line-collimated Kratky-type SWAXS instrument. In contrast to uniform illumination or small-beam approximations, the transmission factor is averaged over the finite illuminated region and weighted by the experimentally measured primary beam profiles. Additionally, we explicitly account for attenuation in the quartz capillary wall and use a full three-dimensional correction model. These features become particularly important for highly absorbing samples. To the best of our knowledge, this specific combination of constrained illuminated volume, capillary wall attenuation and experimentally measured beam profile weighting has not previously been implemented for absolute-scale line-collimated SWAXS data reduction in thin-walled capillaries.

**Figure 2:**
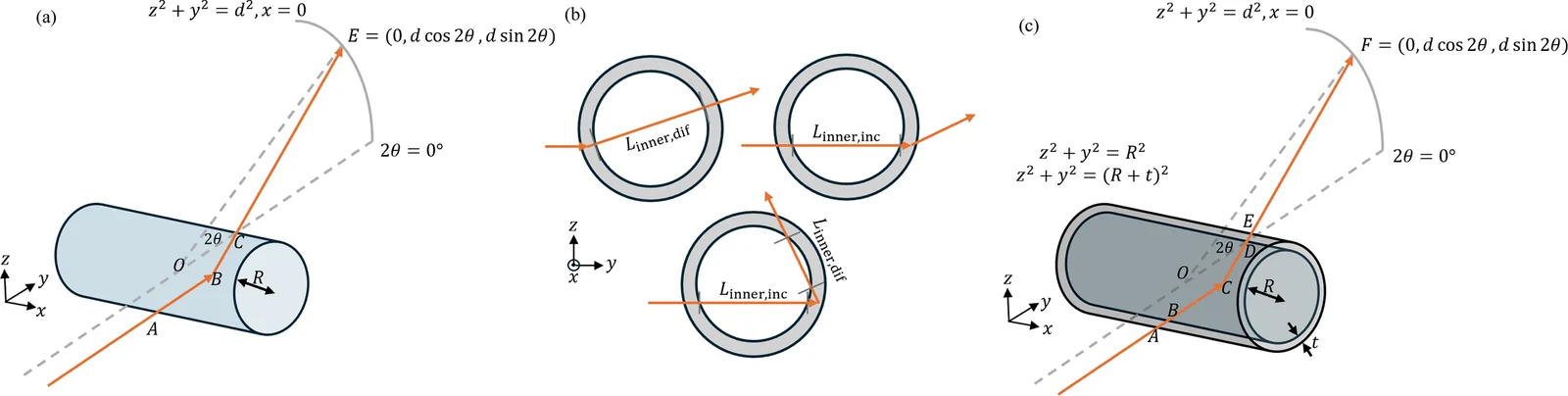
Visualization of the sample and detector geometry considered in our derivation of the path lengths, (*a*) without considering the capillary wall absorption and scattering, (*b*) considering only the scattering and absorption in the capillary wall, and (*c*) considering scattering inside the sample and absorption in the sample and capillary wall.

The experimental geometry is defined in a Cartesian coordinate system with the primary beam propagating along the *y* axis, the horizontal direction along the *x* axis and the vertical direction along the *z* axis. A schematic representation of this geometry, including the sample, the capillary wall and the detector arrangement, is shown in Fig. 2 ▸(*a*).

The scattering-angle-dependent absorption correction is applied directly to the measured raw scattering intensity *I*_raw_(2θ), analogously to equation (1), using the relation
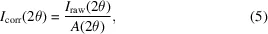
where 2θ is the scattering angle, *I*_corr_(2θ) is the corrected scattering intensity, and *A*(2θ) is the angle-dependent transmission factor calculated for the relevant absorbing material and experimental geometry. The geometric meaning of this correction is clarified by the definition of the transmission factor *A*(2θ), introduced in the following. In a general 3D geometry, the transmission factor can be expressed as a volume integral over the illuminated part of the sample (and its container, if included among attenuation contributions), accounting for attenuation along the incident path to the scattering point and along the scattered path from that point to the detector:
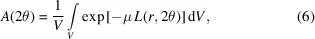
where μ is the linear attenuation coefficient of the material, *L*(*r*, 2θ) is the X-ray path length through the material at a scattering angle 2θ and *V* is the illuminated volume. Equation (6) is essentially the Beer–Lambert attenuation law averaged over all the corresponding incident and exit X-ray paths relevant for the detected scattering at each 2θ. Accordingly, *A*(2θ) represents the average transmission coefficient defined solely for photons contributing to the detected scattering intensity at a given 2θ. For SAXS data, it is often even more natural to express the angular dependence in terms of the length of the scattering vector *q*, defined as
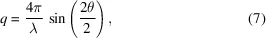
where λ is the wavelength of the X-rays. To facilitate comparison with previous absorption correction studies, we compute and present the correction primarily as a function of the scattering angle 2θ, but also provide scattering-angle-dependent transmission correction factors *A*(*q*) where relevant (see Table S3 in Appendix 7).

Note that the wavelength of the X-rays does not enter this absorption correction formulation explicitly. Its influence on the absorption correction coefficients is fully accounted for through the wavelength dependence of the linear attenuation coefficient μ, which is treated as an input material parameter (Hubbell & Seltzer, 2004 ▸).

**Figure 3:**
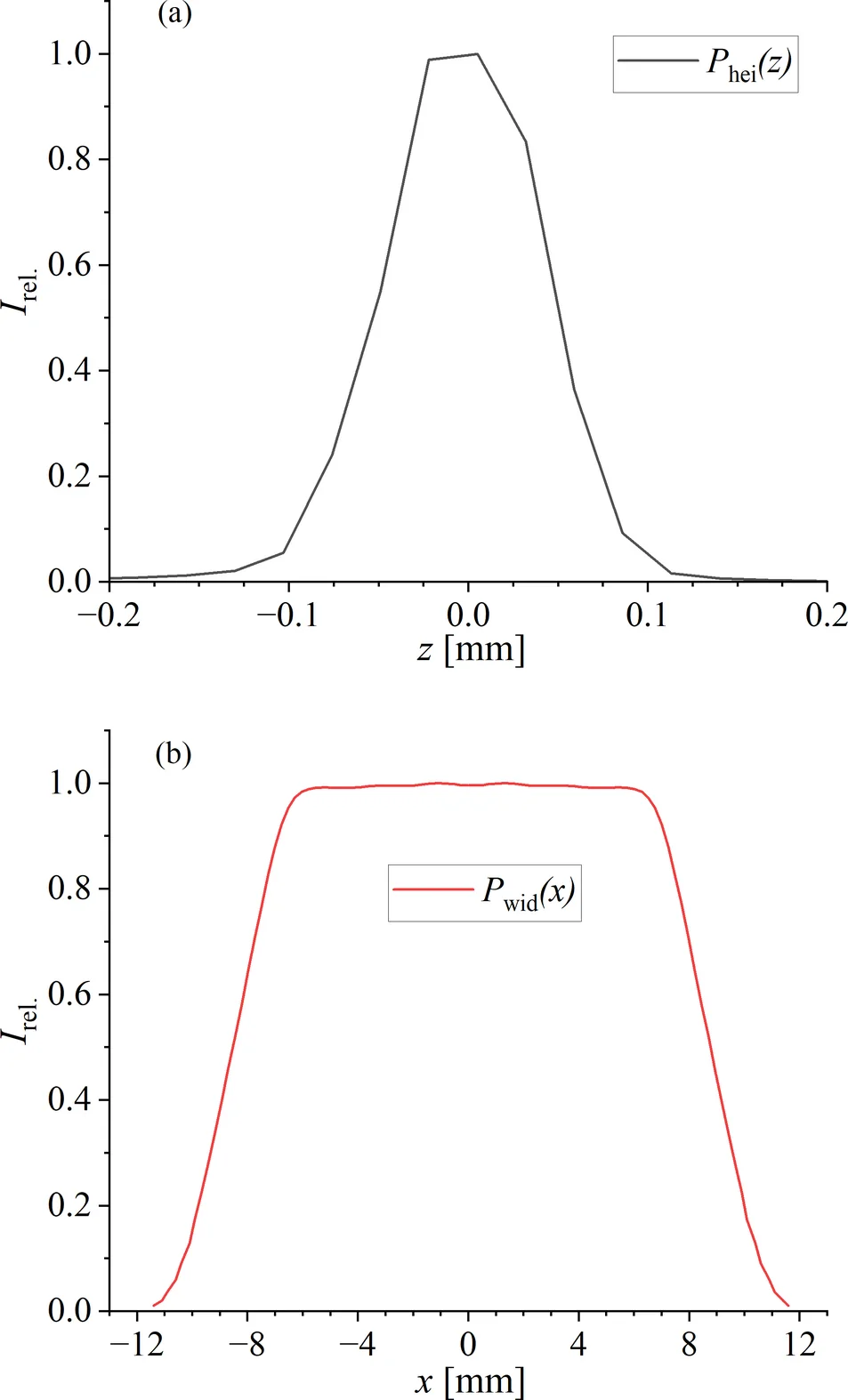
The experimentally obtained (*a*) height *P*_hei_(*z*) and (*b*) width *P*_wid_(*x*) profiles of the horizontal line-collimated primary X-ray beam in our SWAXS setup.

In our SWAXS experimental configuration, the incident beam exhibits an experimentally determined non-uniform intensity distribution, which is measured as two orthogonal profiles: (i) a ‘width’ profile *P*_wid_(*x*) along the horizontal axis *x*, and (ii) a ‘height’ profile *P*_hei_(*z*) along the vertical axis *z*, as shown in Fig. 3 ▸. These two profiles are incorporated into the absorption correction scheme through the corresponding dimensionless weighting function *W*(*x*, *z*). The latter is independent of the beam-propagation coordinate *y* and is defined for the primary beam path lengths through the capillary with the sample as
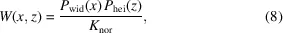
where the normalization factor is given by
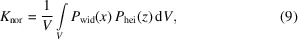
ensuring that
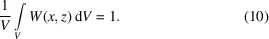


The transmission coefficient *A*(2θ), which accounts for attenuation in both the capillary wall and the sample, is then calculated as
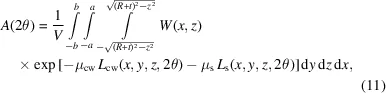
where *V* is the constrained illuminated volume defined by the limits of integration, μ_cw_ and μ_s_ are the attenuation coefficients of the capillary wall and the sample, respectively, and *L*_cw_(*x*, *y*, *z*, 2θ) and *L*_s_(*x*, *y*, *z*, 2θ) denote the corresponding path lengths contributing to the scattering at an angle 2θ. In these path-length terms, the coordinate *y* denotes that the scattering event may occur at any position along the *y* axis, including within the capillary wall, as depicted schematically in Fig. 2 ▸(*b*).

The cylindrical capillary has an inner radius *R* and a wall thickness *t*. It is illuminated non-uniformly, with the illumination represented by *W*(*x*, *z*) over the constrained cross-section region *z* ∈ [−*a*, *a*] and *x* ∈ [−*b*, *b*], while the primary beam propagates along the *y* axis. The limits *a* and *b* define the constrained illuminated scattering volume cross section used in the numerical integration and were chosen from the measured extent of the experimental beam profiles, *i.e.* from the regions where their intensities remain significant. A scattering event occurs at a point *C*(*x*, *y*, *z*) within the constrained scattering volume – either within the capillary wall or within the sample [Fig. 2 ▸(*c*)]. Due to the non-uniform illumination of the scattering volume, the corresponding path lengths must be weighted by the actual local intensity of the primary beam to obtain the weighted average transmission factor in equation (11). To keep the geometric description consistent with the actual experimental setup used in this work, the detector is positioned vertically at a distance *d* from the central capillary axis and is curved such that the detector points lie on the cylindrical surface:
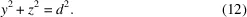


The path length through the sample, *L*_s_(*x*, *y*, *z*, 2θ), is [Fig. 2 ▸(*c*)]
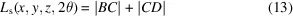
and the total path through the capillary wall, *L*_ew_(*x*, *y*, *z*, 2θ), is
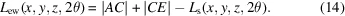


Given the general coordinates of point 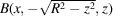, the magnitude of the incident segment inside the sample is
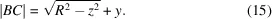


For the scattered beam path length towards the detector, |*CD*|, we consider the ray from point *C* in the direction towards the detector point at the scattering angle of 2θ,
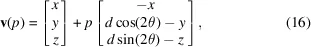
which intersects the inner cylinder *y*^2^ + *z*^2^ = *R*^2^ of the sample. This yields the quadratic equation for parameter *p*,
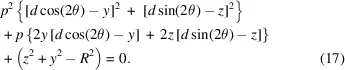


Of the two roots, the larger, *p*_+_, corresponds to the sample exit point, giving the magnitude
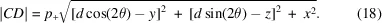
The magnitudes of |*AC*| and |*CE*| are computed analogously using the outer radius (*R* + *t*).

For scattering events occurring within the wall,
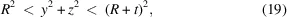
the beam segments through the inner sample cylinder must be treated conditionally: (i) if *y* > 0 and |*z*| < *R*, the incident beam intersects the inner surface, yielding
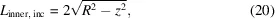
but (ii) if two positive roots *p*_+,inner_ > *p*_−,inner_ exist for the scattered ray intersecting the inner sample cylinder, then
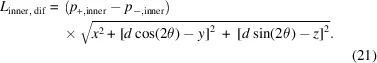


In summary, we note that, in the present context, the transmission factor *A*(2θ) is not a general material property but rather a numerically determined geometry-specific quantity. It is used here as a calculated, angle-dependent and geometry-specific Beer–Lambert transmission factor for the detected scattering intensity, not as an experimentally measured direct-beam transmission factor. It effectively encapsulates information about the specific experimental configuration of the instrument – derived from the primary beam profiles – as well as information on the sample material through its linear attenuation coefficient. In our case, this corresponds to a Kratky-type line-collimated SWAXS setup equipped with a focusing Göbel mirror. In the calculations, only two absorption contributions are considered: (i) attenuation of the X-ray beam within the capillary up to the position of the scattering event, and (ii) attenuation of the scattered ray along its exit path through the remaining capillary material towards the detector. Consequently, the transmission factor *A*(2θ) accounts exclusively for these two contributions and does not include additional instrumental effects such as detector response, multiple scattering or spectral changes in the incident beam. In a practical sense, *A*(2θ) represents a weighted average over all individual path-length contributions arising from the different possible scattering positions within the illuminated scattering volume.

## 3. Experimental and computational methods

Full details of the materials, SWAXS measurements, mol­ecular dynamics simulations and calculation of the angle-dependent transmission factor *A*(2θ) using the developed Python software are provided in Appendix 6 (Glatter, 2018 ▸; Jorgensen *et al.*, 1996 ▸; Watkins & Jorgensen, 2001 ▸; Dodda *et al.*, 2017 ▸; Jorgensen *et al.*, 1983 ▸; Abascal & Vega, 2005 ▸; Mahoney & Jorgensen, 2000 ▸; Berendsen *et al.*, 1987 ▸; Abraham *et al.*, 2024 ▸).

## 4. Results and discussion

As a starting point for treating absorption correction in our Kratky-type SAXS/SWAXS instrument, we used the work of Bowden & Ryan (2010 ▸), since their approach is conceptually closest to the capillary geometry considered here. In their terminology, the relevant case corresponds to a cylindrical specimen inside an annular support, treated in powder-diffraction capillary geometries. We adapt this geometry to the one shown in Fig. 1 ▸, which is commonly used in SAXS and SWAXS experiments on liquid systems contained in thin-walled cylindrical capillaries, as is typical in colloidal and soft-matter research. Although Bowden and Ryan considered an incident beam smaller than the specimen diameter and offset from the cylindrical axis, they discuss this aspect only in relation to 2θ-dependent offsets in experimental peak positions in their specific powder-diffraction geometry (Bowden & Ryan, 2010 ▸). However, we explicitly define and analyse the constrained illuminated scattering volume for the symmetric capillary alignment typical of the Kratky-type geometry to study its effect on the transmission correction factor in a systematic way. This is introduced through the integration limits *a* and *b* in equation (11), where *a* defines the vertical half-height of the constrained illuminated volume and *b* defines the horizontal half-width along the capillary axis.

**Figure 4:**
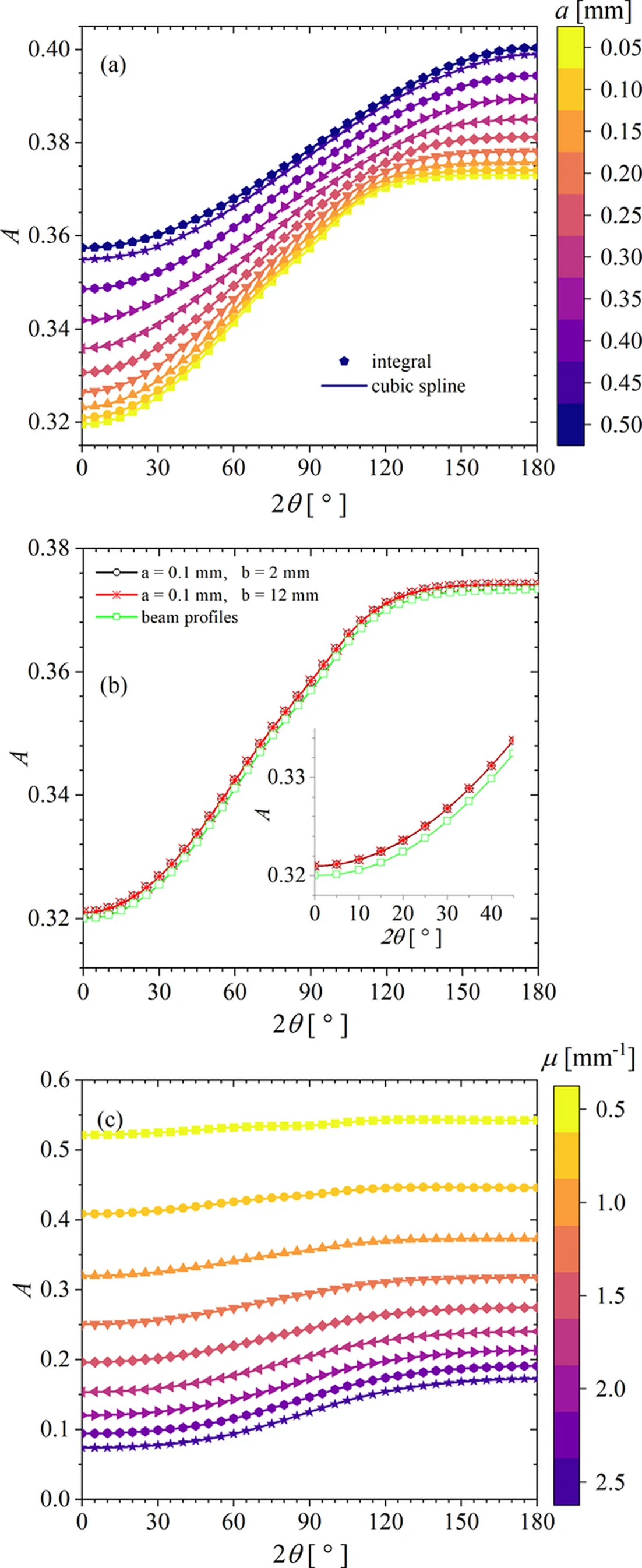
(*a*) The effect of increasing the primary beam half-height *a* at constant beam half-width *b* = 12 mm, capillary-wall thickness *t* = 0.01 mm and linear attenuation coefficient μ = 1.0 mm^−1^. (*b*) The effect of changing the beam half-width *b* and including the experimental beam profiles, all at constant *t* = 0.01 mm and μ = 1.0 mm^−1^. (*c*) The effect of increasing the linear attenuation coefficient μ on the angular dependence of the transmission coefficient *A*(2θ) at constant *a* = 0.1 mm, *b* = 12 mm and *t* = 0.01 mm.

To gain a better understanding of the practical consequences of this adaptation, we first examined how the calculated transmission coefficient *A*(2θ) changes when the main geometric and material parameters of the model are varied separately. The effect of varying the beam half-height *a* in the vertical *z* direction is analysed systematically in Fig. 4 ▸(*a*) for a constant beam half-width *b* = 12 mm and uniform central illumination of the capillary. Overall, it is evident that increasing the vertical beam height results in a higher transmission coefficient at a given scattering angle. This is because illuminating a larger proportion of the capillary in the *z* direction increases the fraction of shorter path lengths, which contribute less to the total attenuation. These results highlight that the beam height has a significant influence on the transmission coefficient. Therefore, it is crucial to determine it carefully and include it as realistically as possible in the calculation of the coefficient *A*(2θ).

The effect of varying the beam half-width *b* from 2 to 12 mm for a constant beam half-height *a* = 0.1 mm is presented in Fig. 4 ▸(*b*). The two curves (black open circles and red crosses) practically coincide, indicating that the horizontal width of the primary beam has almost no detectable effect on the transmission coefficient. Note that, although variations in the primary beam profile in the horizontal plane strongly affect the projection of scattering intensity onto the detector, leading to considerable instrumental smearing effects in the SAXS regime (Glatter, 2018 ▸), they evidently have only a negligible influence on the absorption correction. This is because, for fixed material attenuation coefficients, the absorption depends directly on the path length of the X-ray beam within the absorbing material, *i.e.* the sample and the capillary wall. Since the incident beam propagates along the *y* axis, displacements of the scattering event along the capillary axis (*x*) do not change the incident path length through this material. They affect only the exit path, and even this effect is small because the sample-to-detector distance (267 mm in our setup) is much larger than the horizontal displacement within the illuminated beam. The corresponding change in the total path length within the absorbing material is therefore small and has a barely noticeable effect on the absorption correction, as demonstrated in Fig. 4 ▸(*b*). To test this point further, we also considered the limiting case where the primary beam width is reduced to values comparable to the beam height, as may occur in experimental setups with strongly reduced slit width; an example with *a* = *b* = 0.1 mm is provided in Section 8.1 of Appendix 8.

The green curve with open squares in Fig. 4 ▸(*b*) represents a key result of the presented extension of the absorption correction procedure. It shows the *A*(2θ) data obtained by applying both experimentally measured primary beam profiles as weights within the constrained illuminated scattering volume, while also accounting for the capillary-wall attenuation. The fact that this curve does not deviate substantially from the other two curves in Fig. 4 ▸(*b*) may be somewhat misleading when assessing the importance of this extension. However, this only means that the selected value *a* = 0.1 mm is rather close to the appropriate effective primary beam half-height in our Kratky-type camera.

The message of Fig. 4 ▸(*b*) is one of the most important messages of this study and is twofold. First, properly accounting for the vertical illumination geometry of the primary beam is crucial for absorption correction, and including the non-uniform experimentally measured height profile of the primary beam in the correction procedure is the most reliable way to do this. Second, even when only a uniform primary beam is considered, there exists an effective beam half-height value that can closely approximate the effect of the primary beam height on *A*(2θ). However, this effective value is not known *a priori* and would have to be estimated correctly. The most realistic solution is therefore to incorporate the experimentally obtained incident beam profiles directly into the procedure, as implemented through the weighting function *W*(*x*, *z*) in equation (11).

Having established this key geometrical point, we next consider the material parameter entering the correction. Therefore, Fig. 4 ▸(*c*) illustrates the effects of increasing the linear attenuation coefficient of the sample. Higher attenuation not only reduces the overall transmission but also enhances the angular dependence of the transmission coefficient. The latter occurs because increasing μ amplifies the differences between the contributions from different path lengths within the illuminated scattering volume. At small scattering angles, the average total path length through the absorbing material remains similar to the corresponding forward-transmission path length, whereas at larger angles the distribution of total path lengths broadens. Correspondingly, over the scattering angle range up to about 50° in Fig. 4 ▸(*c*) – roughly matching the SWAXS range of our Kratky-type instrument – this effect on the angular dependence is still moderate but becomes increasingly pronounced at higher angles.

The numerical reliability of the numerical integration in equation (11) and geometrical robustness of the procedure are examined further in Appendix 8. The root-mean-squared error of *A*(2θ) falls below 0.1% for all tested attenuation coefficients, while the influence of capillary conicity was found to be negligible even for unrealistically large conicities.

In practice, the purpose of the proposed procedure is to correct measured SWAXS curves only over the measured angular range from 2θ_min_ to 2θ_max_. Therefore, only the calculated *A*(2θ) values within this range are required in equation (5), thereby avoiding the practical problems associated with primary beam transmission measurements discussed in Appendix 3. Nevertheless, it is useful to relate the calculated *A*(2θ) to the scalar factor *T*_*j*_ from equation (1). In equation (11), the Beer–Lambert attenuation is averaged over the incident and exit paths of the photons that contribute to the detected elastically scattered intensity at a given 2θ. The forward-scattering limit *A*(0) in equation (11) follows from the same reasoning. Nonetheless, the photon paths in the forward-scattering limit are the same as those of the directly transmitted photons recorded in primary beam transmission measurements. Therefore, *A*(0) could also be applied consistently to correct *I*_*j*,raw_(0) from equation (1), provided that the latter can be measured reliably (Appendix 3).

Consequently, for weakly absorbing samples with negligible angular dependence of the absorption correction, *A*(0) can be used as an approximate scalar numerical correction factor, analogously to *T*_*j*_ in equation (1). This is a useful qualitative alternative when *T*_*j*_ cannot be measured reliably. It also raises the question of whether *A*(0) could be approximated even more simply and still provide a satisfactory qualitative approximation. A straightforward option would be to use a deliberately simplified numerical correction based on the Beer–Lambert attenuation law, which considers only the sample and capillary-wall thicknesses and a single effective path length for all photons (Appendix 9).

**Figure 5:**
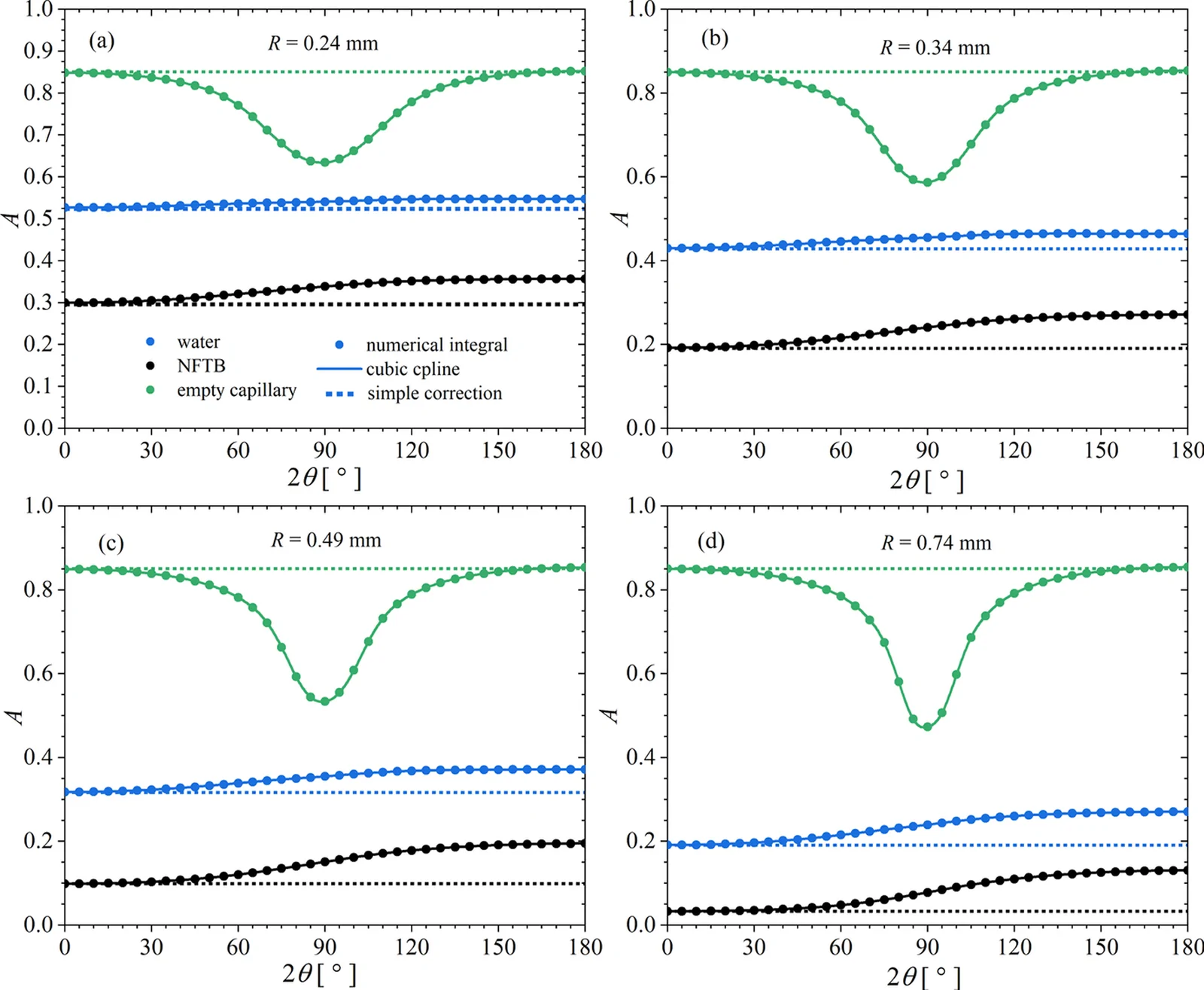
The angular dependence of the transmission coefficient *A*(2θ) at constant *a* = 0.1 mm, *b* = 12 mm, *t* = 0.01 mm, μ_quartz_ = 8.099 mm^−1^, μ_water_ = 1.01 mm^−1^ and μ_NFTB_ = 2.20 mm^−1^ for the empty capillary (green solid line), water (blue solid line) and NFTB (black solid line) compared with the corresponding simplistic model values (dotted lines) for capillary radii of (*a*) 0.24 mm, (*b*) 0.34 mm, (*c*) 0.49 mm and (*d*) 0.74 mm.

We evaluate this simplified scalar correction strategy against the full *A*(2θ) transmission coefficients in Fig. 5 ▸. It shows the *A*(2θ) values (full lines with symbols) together with the corresponding simplified Beer–Lambert scalar estimates (horizontal dotted lines) for the empty capillary, water and NFTB, for several capillary radii. The simplified Beer–Lambert scalar estimate is not physically equivalent to the full angle-dependent correction because it neglects the finite illuminated volume, beam-profile weighting and angle-dependent exit paths. Nevertheless, its values remain very close to the corresponding *A*(0) values for all tested cases, indicating that this similarity is not an artefact of the selected capillary radius. At larger scattering angles, however, the full *A*(2θ) curves increasingly deviate from this scalar value.

Fig. 5 ▸ therefore supports two very important practical conclusions. First, the simplified Beer–Lambert-type scalar transmission estimate is a qualitatively useful approximation when the angular dependence of the transmission function is negligible, but it cannot replace the full angle-dependent correction when quantitative SWAXS data reduction is required. Second, the transmission functions for water and the empty capillary differ substantially, both in their absolute transmission level and in their angular dependence. This indicates that the water/empty-capillary correction step must be executed with care. If unreliable experimental transmission factors are used, this step may introduce notable errors into absolute-scale calibration using water as a secondary standard.

The practical consequences of these correction strategies are briefly summarized here and documented in detail in the supporting information. Appendix 4 first shows the failure of the conventional scalar primary beam transmission correction for the strongly absorbing NFTB sample. Section 10.1 of Appendix 10 then shows a successful SWAXS data reduction for this sample using the proposed numerical correction procedure. The water/empty-capillary correction relevant to absolute-scale calibration is presented and discussed in Section 10.2 of Appendix 10, a diverse range of Brij 35/alcohol/water systems in Section 10.3, and the simplified Beer–Lambert-type scalar approximation in Section 10.4. Appendix 11 provides further direct experimental cross checks of the proposed correction procedure for the water and NFTB datasets. These checks use independent Mythen 1K SAXS detector measurements performed on the same sample, in the same capillary and on the same Kratky-type camera, without changing the sample/capillary arrangement. Together, these examples and cross checks support the practical applicability of the calculated transmission workflow for background subtraction and absolute scaling in the experimental cases considered here.

## 5. Conclusions

The central result of this study is that, when experimentally determined scalar primary beam transmission factors become unreliable, the corresponding transmission correction problem can be bypassed using calculated geometry-specific transmission factors *A*(2θ) based only on the experimental geometry, measured primary beam profiles and tabulated attenuation coefficients. For the line-collimated Kratky-type SWAXS geometry considered here, the vertical illumination profile is the key geometric parameter controlling the absorption correction, whereas the horizontal beam width effect is negligible. Using the measured primary beam profiles removes the need to choose an effective uniform beam height as an additional approximation. The performance of the proposed angle-dependent transmission correction procedure has been demonstrated for the strongly absorbing NFTB sample. For this sample, the conventional scalar transmission correction led to a physically inconsistent result after background subtraction, while the numerical correction resolved this problem and enabled physically meaningful absolute-scale SWAXS data reduction.

The results also show that the bias in experimentally determined scalar transmission factors associated with primary beam radiation hardening is not limited to strongly absorbing samples. Such bias can become important whenever a measurement series contains samples with substantially different X-ray attenuation. One example is the water/empty-capillary measurement pair used in routine absolute scaling with water as a secondary standard. The proposed correction procedure successfully bypasses the corresponding transmission correction problem in this absolute-scaling step as well. Comparisons of independent model-based theoretical SWAXS curves (Lajovic *et al.*, 2010 ▸) with the reduced experimental data, and additional measurements with the Mythen 1K detector, provide consistency checks of the corrected data-reduction procedure. Importantly, neither the correction factors nor the corrected experimental SWAXS curves were fitted, tuned or adjusted to improve the agreement between the compared data.

Taken together, the presented results suggest the following practical hierarchy of correction strategies: (i) conventional primary beam transmission correction is adequate when the measured transmission factors *T*_*j*_ are reliable and absorption differences between measurements are small; (ii) a simplified Beer–Lambert-based scalar correction can be useful as a reasonably fast qualitative or approximate correction when the angular dependence is weak; and (iii) the full *A*(2θ) correction is required for physically consistent quantitative SWAXS analysis when absorption differences between measurements are significant. This practical distinction is not specific to the Kratky-type geometry considered here and is relevant more generally whenever SAXS/SWAXS data reduction relies on experimentally determined scalar transmission factors whose reliability cannot be ensured.

## 6. Related literature

For further literature related to the supporting information, see Merck Life Science (2025 ▸), Nürnberg *et al.* (2016 ▸), Prince (2004 ▸), Rodriguez *et al.* (2001 ▸) and Tanaka *et al.* (2001 ▸).

## Supplementary Material

Additional information with extra figures and tables. DOI: 10.1107/S1600576726006837/jl5129sup1.pdf

ZIP file of data. DOI: 10.1107/S1600576726006837/jl5129sup2.zip

## Data Availability

The experimental data reported in this article are provided as a zip archive in the supporting information.
