# Supplementary material for: Angle-dependent X-ray absorption correction in small- and wide-angle X-ray scattering: accounting for constrained scattering volume, beam profiles and capillary wall attenuation
Source: J Appl Crystallogr. 2026 Aug 6;59(Pt 5):1374–83. doi: 10.1107/S1600576726006837 (PMC13636045; doi:10.1107/S1600576726006837)
Supplement: Supplementary file 1 [file j-59-01374-sup1.pdf]

JOURNAL OF APPLIED CRYSTALLOGRAPHY

Volume 59 (2026)

Supporting information for article:

Angle-dependent X-ray absorption correction in small- and wide-angle X-ray scattering: accounting for constrained scattering volume, beam profiles and capillary wall attenuation

Jure Kovač, Andrej Jamnik, István Szilágyi and Matija Tomšič

Supporting information for:

Angle-dependent X-Ray Absorption Correction in SWAXS: Accounting for Constrained Scattering Volume, Beam Profiles and Capillary Wall Attenuation

Authors

Jure Kovač[a], Andrej Jamnik[a], István Szilágyi[b] and Matija Tomšič[a]*

[a]Faculty of Chemistry and Chemical Technology, University of Ljubljana, Večna pot 113, Ljubljana, 1000, Slovenia

[b]MTA-SZTE Momentum Biocolloids Research Group, Department of Physical Chemistry and Materials Science, Interdisciplinary Research Center, University of Szeged, Szeged, Hungary, H-6720

Correspondence email: matija.tomsic@fkkt.uni-lj.si

Supporting information provides additional experimental, geometrical, and computational details and the public link to the Python program developed to calculate the angle-dependent absorption correction factor for SWAXS measurements in horizontal line-collimated geometry, which is: https://github.com/jurekovac340/SWAXS-absorption-correction.

Table of Contents

Appendix 1 – Background Motivation and Scope 2
Appendix 2 – SAXS/SWAXS Measurements and Conventional Data Reduction 2
Appendix 3 – Primary-Beam Radiation Hardening in SWAXS 3
Appendix 4 – Conventional Reduction of SWAXS Data for the NFTB Sample 5
Appendix 5 – Numerical Absorption Correction Approaches in XRD and SWAXS 6
Appendix 6 – Experimental and Computational Methods 7
6.1 Materials 7
6.2 SWAXS Measurements 8
6.3 Molecular Dynamics Simulations and Scattering Intensity Calculations 8
6.4 Computation of Angle-Dependent Transmission Correction Factors 9
Appendix 7 – Attenuation Coefficients $A(\mu, 2\theta)$ and Interpolation Tables 10
Appendix 8 – Additional Geometrical and Numerical Checks 13
8.1 Comparable Primary-Beam Height and Width 13
8.2 Convergence of the Numerical Integration in Equation (11) 15
8.3 Effect of Capillary Conicity 15
Appendix 9 – Simplified Beer-Lambert-Type Scalar Transmission Estimate 16
Appendix 10 –Absorption Correction Examples and Absolute Scaling 17
10.1 Reduced NFTB and Water SWAXS Data 17
10.2 Exploring Water/Empty-Capillary Scaling Bias 18
10.3 Other Reduced SWAXS Data Examples 20
10.4 Simplified Beer-Lambert Estimate Example and Practical Implications 22
Appendix 11 – Experimental Mythen 1K Detector Cross-Check 22

Appendix 1 – Background Motivation and Scope

The motivation for the present absorption-correction study originates from our broader use of SWAXS as a quantitative tool for studying liquid and soft-matter molecular and colloidal systems on an absolute intensity scale. It arose particularly from our recent studies of highly fluorinated per- and polyfluoroalkyl substances (PFAS), which represent an important class of environmentally and technologically relevant systems and for which X-ray attenuation can be substantial. This motivation is closely connected with our use of the so-called "complemented-system approach" (Cerar *et al.*, 2020, Tomšič, Jamnik*, et al.*, 2007, Lajovic *et al.*, 2010), where molecular simulations and theoretical calculations of SWAXS intensities are used to validate the molecular model and to interpret the experimental SWAXS data in greater detail. In such studies, reliable background subtraction and absolute scaling are essential, because any systematic errors directly affect the comparison between experimental and calculated SWAXS data.

In our previous SWAXS studies, the conventional scalar transmission correction performed sufficiently well because the attenuation of samples within a given series did not differ substantially (Cerar *et al.*, 2021, Cerar *et al.*, 2020, Cerar *et al.*, 2019, Tomšič *et al.*, 2019, Tomšič *et al.*, 2018, Cerar *et al.*, 2017, Lajovic *et al.*, 2012, Lajovic *et al.*, 2009, Tomšič, Jamnik*, et al.*, 2007, Tomšič, Vlcek*, et al.*, 2007, Lajovic *et al.*, 2010). The problem became apparent only when we turned to highly fluorinated PFAS-related systems, where we encountered severe problems when applying the conventional basic SWAXS data-reduction procedures, which led to distorted or non-physical scattering profiles. The highly fluorinated alcohol nonafluorotert-butanol (NFTB) can be taken as a representative example of such problems. These problems were not merely a technical inconvenience for us, but a direct obstacle to obtaining physically meaningful absolute-scale SWAXS data for systems of current interest, including PFAS-related liquids. Developing a suitable numerical absorption correction therefore appeared to be the most viable option.

Application of the numerical correction procedure proposed in this paper showed that the conventional correction can introduce a slight but systematic error even in the routine correction step needed for absolute scaling using water as a secondary standard. This arises from a notable difference in X-ray absorption between the water and empty-capillary measurements. We demonstrated this effect using calculated theoretical SWAXS curves and experimental water SWAXS data. We also reprocessed a few selected chemically diverse experimental examples from our recent study on the nonionic surfactant system Brij 35 in alcohols and water (Cerar *et al.*, 2021). The consistency between the model-based theoretical SWAXS curves and the experimental data improved when the proposed data-reduction procedure was applied. Thus, the practical motivation for the present work is not limited to a single strongly absorbing test sample but extends to the reliability of background subtraction and absolute scaling in routine quantitative SWAXS analysis.

Appendix 2 – SAXS/SWAXS Measurements and Conventional Data Reduction

Equations (1-4) in the main article describe the conventional SAXS/SWAXS workflow of basic data reduction in terms of absorption and background corrections and absolute scaling. In this context, it is important to understand that SAXS or SWAXS experiments typically involve at least two measurements: one of the sample in a quartz capillary, and one of the pure solvent in the same capillary. After applying a basic absorption correction, the solvent signal is subtracted from the sample signal to isolate the excess scattering from the structuring material of interest. This subtraction also removes the capillary's scattering contribution. In addition, a measurement of water in a capillary is often included

together with an empty-capillary measurement to determine the scattering intensity of water at zero angle. If water itself is the solvent, the solvent measurement already serves this purpose. This experimental value is then compared with the theoretical differential scattering cross-section of water at zero angle, yielding a scaling factor that places the entire SAXS or SWAXS profile on an absolute scale using water as a secondary standard – a well-established and widely used procedure described by Orthaber et al. (Orthaber *et al.*, 2000).

It is important to note that, in line-collimated Kratky-type instruments and accompanying software, the basic absorption correction is typically applied as the conventional scalar transmission correction. It uses the maximum height, or integrated area, of the attenuated primary beam measured simultaneously with the scattering data. This correction accounts at the same time for primary-beam absorption in the sample and for temporal variations of the incident flux, without distinguishing between the two contributions. This means that it combines the so-called transmission correction and the flux correction (Pauw, 2013, Orthaber *et al.*, 2000, Bergmann *et al.*, 2000). Specifically, the attenuated beam transmitted through the capillary containing an individual sample is measured at zero scattering angle alongside the corresponding angle-dependent scattering of the sample on the detector during the whole measurement time. In practice, this primary-beam peak also serves to identify the zero-angle scattering position, i.e., the point on the detector corresponding to a scattering vector of zero.

This fully experimental conventional scalar transmission correction is performed individually for each sample prior to background and solvent scattering subtraction. It explicitly represents a zero-angle-based transmission correction procedure, in which the transmission factor at $q = 0$ is applied as a single scalar constant to rescale the entire SAXS or SWAXS curve. In this way, the correction is applied uniformly at all scattering angles and no possible angular dependence of absorption is considered. While this approach generally performs well for a series of samples with similar X-ray absorption – as is often the case in dilute liquid or soft-matter systems – it may prove practically inadequate or even fail completely when absorption varies significantly across samples. An example leading to non-physical negative SAXS intensities is shown and discussed later in Appendix 4, while the origin of such problems is discussed in Appendix 3 in terms of primary-beam radiation hardening.

It should be emphasized that the conventional scalar transmission correction can still work well for a series of measurements with very similar attenuation. In such cases, the degree of primary-beam radiation hardening, and therefore the bias in the experimentally determined scalar transmission factors, is expected to be similar for all measured curves. Since the same type of bias then affects the sample and background measurements in approximately the same way, it largely cancels during relative scaling and subtraction. The limitation discussed here becomes important when the attenuation differs substantially between the measurements. In such a case, the corresponding transmission-factor biases are no longer approximately proportional and do not cancel, so they can lead to systematic errors in the conventional reduction workflow.

Appendix 3 – Primary-Beam Radiation Hardening in SWAXS

In a typical Kratky-type laboratory SWAXS instrument with X-ray tube and multilayer optics, the incident beam is not truly monochromatic, even though it is often treated as such. A conventional Cu-anode X-ray tube emits a broad *bremsstrahlung* spectrum with characteristic lines (Cu K_{α} and Cu K_{β}) and the installed multilayer Göbel mirror (Bergmann *et al.*, 2000) is used to select and focus the Cu Kα radiation. In contrast to crystal monochromators, multilayer optics exhibit energy resolutions on the order of about 0.3-3.5 %, meaning that they transmit a finite band of photon energies. Experimentally

measured performance of multilayer optics has shown harmonic contamination, including a reported 17% third-harmonic contribution in one such study (Oberta *et al.*, 2012). Higher harmonic contamination in nominally monochromatic laboratory X-ray beams is also reported by Kirschbaum et al., who note that for Cu radiation, harmonics up to higher diffraction orders may be present (Kirschbaum *et al.*, 1997). They also emphasize that position-sensitive detectors cannot discriminate photon energy (Kirschbaum *et al.*, 1997). This is consistent with a multilayer-mirror review for diffraction and SAXS, which describes graded multilayers as optics that monochromatize and collimate Cu radiation but still leave spectral impurities (Shimizu & Omote, 2008).

In principle, a multilayer mirror reflects X-rays through Bragg interference from multiple interfaces between layers with different refractive indices, arranged with a designed periodic spacing d. Constructive interference of the reflected waves occurs when the Bragg condition is satisfied (Shimizu & Omote, 2008):

$$n\lambda = 2d \sin\alpha \tag{S1}$$

where $n \in \mathbb{Z}^+$, λ is the wavelength, d is the multilayer period, and α is the grazing incidence angle. For a given d and α, this equation admits multiple solutions:

$$\lambda_n = \frac{2d \sin\alpha}{n} \tag{S2}$$

Thus, if the mirror is aligned to reflect radiation of wavelength λ_1 (eg. Cu K_α), it may also satisfy the Bragg condition for higher-order harmonics present in the emission spectrum of the X-ray tube $\lambda_n = \lambda_1/n$, corresponding to photons of higher energy $E_n = nE_1$, leading to (high-energy) contamination of the primary beam.

Once such a non-ideally monochromatic beam passes through an absorbing material (e.g. a strongly absorbing sample or a semi-transparent beamstop), primary-beam radiation hardening can occur (Baur *et al.*, 2019, Brooks & Di Chiro, 1976, Van de Casteele *et al.*, 2002, Hövelmann *et al.*, 2019, Wu & Li, 2024). The attenuation coefficient of the material $\mu(E)$ generally decreases with increasing photon energy. Therefore, low-energy photons are attenuated more strongly than high-energy photons, causing the relative fraction of higher-energy photons to increase in the primary beam transmitted through the sample. Such a spectral change is referred to as radiation hardening. Consequently, the transmitted primary-beam intensity cannot be accurately described by a single Beer–Lambert attenuation factor with a constant μ. Instead, it should be treated as a spectral average, in which each photon energy is attenuated according to $\mu(E)$ and weighted by the source spectrum $N(E)$ and detector sensitivity $S(E)$ (Baur *et al.*, 2019):

$$I(x) \propto \int N(E) e^{-\mu(E)x} S(E)\, dE \tag{S3}$$

In many SAXS and SWAXS instruments, a semi-transparent beamstop is installed to prevent the primary beam from overloading the sensitive detector. At the same time, it allows the primary beam to be recorded for normalization, i.e. transmission and flux correction, and for determining the q-scale origin. The experimentally measured attenuated primary-beam peak is then used to determine a scalar transmission factor for the whole scattering curve (see transmission factor T_j in equation (1) in the paper). This is precisely where radiation hardening becomes relevant for the conventional transmission correction. The primary beam is first attenuated by transmission through the sample and then further through the semi-transparent beamstop, which additionally spectrally suppresses the lower-energy part of the spectrum. In such a case, the measured transmission is correspondingly overestimated relative to the transmission of a truly monochromatic beam. Especially when the measurements being compared differ substantially in X-ray attenuation, the degree of radiation hardening can also differ notably. This

can lead to systematic errors in absorption correction, background subtraction and absolute intensity scaling of SAXS and SWAXS data.

This interpretation is also supported by recent literature on harmonic suppression, which shows that higher-order harmonic contamination can remain relevant in practical X-ray optical systems. Wu and Li demonstrated that, even after monochromatization at a synchrotron SAXS beamline, the incident beam may need to be treated as a mixture of fundamental and higher-order components when the direct beam is strongly attenuated by a semi-transparent beamstop (Wu & Li, 2024). Similar concerns regarding Bragg-allowed higher-order harmonics and their suppression have been discussed for synchrotron beamline optics and monochromators (Polikarpov *et al.*, 2014, Chen *et al.*, 2018). These studies therefore support the conclusion that monochromatization alone does not guarantee complete spectral purity and that residual higher-energy components can bias transmission-based measurements when the direct beam is strongly attenuated.

Appendix 4 – Conventional Reduction of SWAXS Data for the NFTB Sample

This appendix illustrates the practical failure of the conventional scalar primary-beam transmission correction for the strongly absorbing NFTB sample. The general reduction workflow is described in Appendix 2, while the origin of the primary-beam transmission bias associated with radiation hardening in a Kratky-type setup is discussed in Appendix 3. Here, we focus on the resulting data-reduction sequence for NFTB, water and the empty capillary, and show how the conventional experimental transmission correction leads to a non-physical result for NFTB.

The liquid samples are measured in thin-walled quartz capillaries, while an empty capillary is measured separately to determine the capillary background contribution that must be subtracted during data reduction. The corresponding raw experimental SWAXS profiles of NFTB in a capillary, water in a capillary and the empty capillary, obtained directly from the imaging-plate readout without any further processing, are shown in Figure S1a. Thus, all three profiles contain the capillary contribution, whereas the NFTB and water measurements also contain scattering from the liquid. At lower q values, the ordering of the profiles still partly reflects this additional liquid scattering contribution. However, the effect of X-ray absorption is also clear: the measured intensity of the empty capillary (green circles) becomes comparable to, or even higher than, that of the strongly absorbing NFTB sample (black circles). In part of the q-range, it also becomes comparable to that of water (blue circles). This reflects the considerably higher transmission of the empty capillary compared with the sample-filled capillaries, which attenuate the primary beam more strongly and therefore show an overall reduction in the measured raw intensity.

These data were then corrected using the conventional experimental scalar primary-beam transmission procedure, i.e., by normalizing each profile over the entire q-range to the corresponding attenuated primary-beam intensity measured simultaneously with the scattering data (Pauw, 2013, Orthaber *et al.*, 2000). The corresponding results are presented in Figure S1b. In part of the q -range, the scattering intensity of the empty capillary (green circles) still exceeds that of the NFTB sample (black circles) – this is a clear indication that the conventional transmission correction is inadequate for this strongly absorbing sample. Specifically, while the normalized capillary scattering can be subtracted from the water data without difficulty, applying the same procedure to NFTB produces non-physical negative intensity values in the capillary-subtracted curve (black open circles in Figure S1c). The same

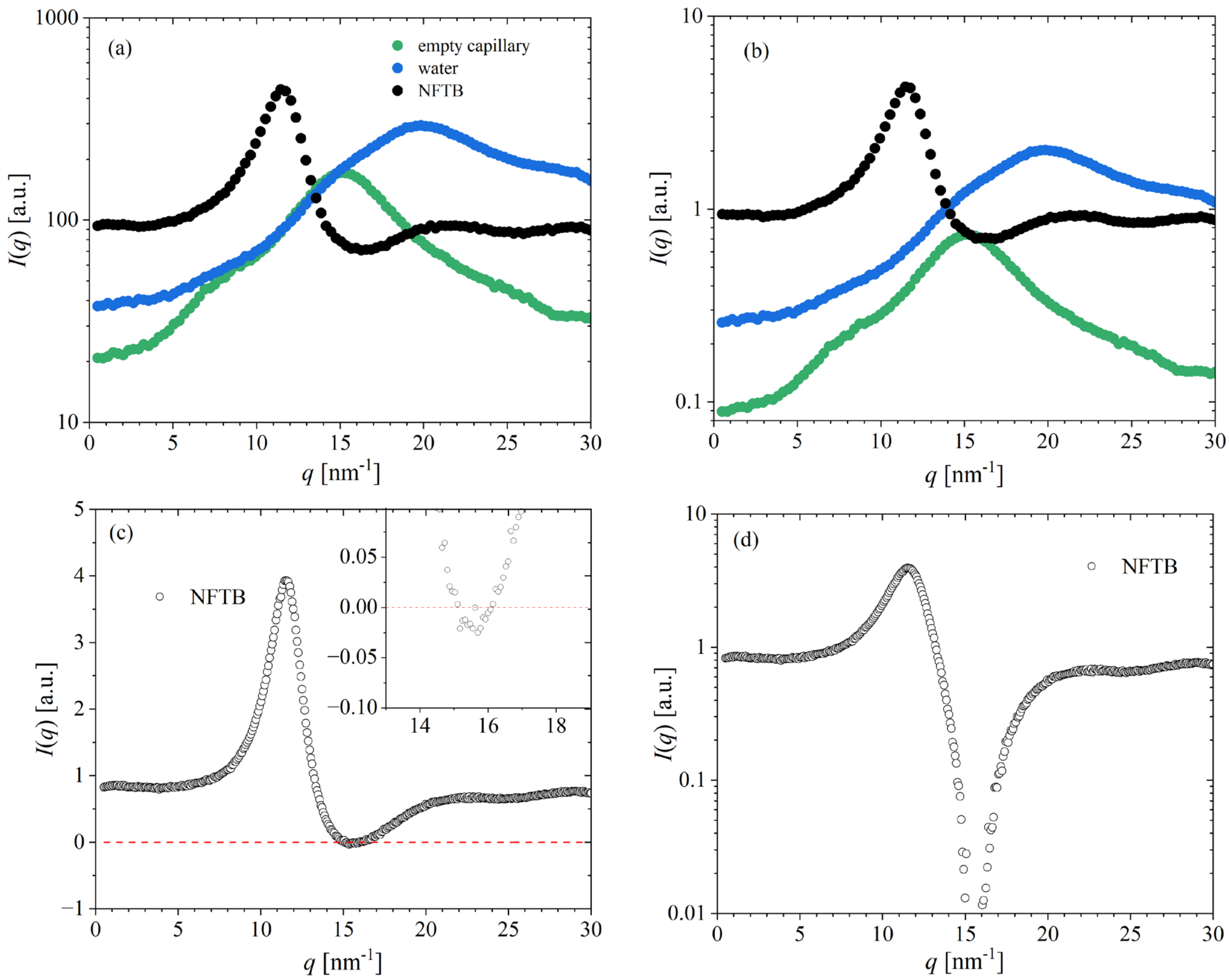

Figure S1. SWAXS data-reduction sequence for the strongly absorbing NFTB sample. (a) Raw experimental SWAXS profiles of NFTB, water and the empty capillary without any normalization. (b) The same profiles after the conventional experimental scalar primary-beam transmission correction. (c) Capillary-subtracted NFTB profile obtained following the conventional correction and shown on a linear intensity scale; the inset magnifies the q-range where non-physical negative values occur. (d) The same capillary-subtracted NFTB profile shown on a logarithmic intensity scale.

curve is shown again in Figure S1d, but on a logarithmic intensity scale. This demonstrates the practical failure of the conventional scalar transmission correction for NFTB. More generally, this behaviour illustrates how conventional transmission-based procedures can fail in strongly absorbing systems when the experimentally measured primary-beam transmission is biased by radiation hardening, leading to an inaccurate transmission correction coefficient.

Appendix 5 – Numerical Absorption Correction Approaches in XRD and SWAXS

Early, foundational approaches to absorption correction in XRD were graphical in nature (Claassen, 1930, Bradley, 1935) and were later advanced by introducing numerical integration approaches (Bond, 1959) yielding the tabulated absorption factors as a function of the product μR (i.e., the linear attenuation coefficient, μ, and the cylinder radius, R). These models were further refined using trigonometric path length expressions to allow for more accurate integration and empirical interpolation

(Dwiggins, 1975, 1972). While initial efforts focused solely on the sample itself, later studies considered absorption in supporting structures such as capillaries or annular holders (Ritter *et al.*, 1951, Møller & Jensen, 1952, Schmitt & Ouladdiaf, 1998).

Building on this foundation, Bowden and Ryan developed an important numerical 2D integration framework that included both the beam path-length contributions through the sample and the container (Bowden & Ryan, 2010), providing sophisticated absorption correction for a range of cylindrical and annular geometries. They highlighted the importance of accounting for absorption in thin-walled annular supports, especially when the attenuation coefficients of the sample and holder are comparable. They also showed that neglecting absorption in the support structure, such as a glass capillary, can lead to significant errors, particularly at low diffraction angles and for samples with high μ. A related pixel-resolved treatment for an infinite cylindrical specimen and a two-dimensional detector was later reported by Sulyanov et al., who showed that absorption in such geometries may vary over the detector surface and cannot always be represented only by an in-plane 2θ dependence (Sulyanov *et al.*, 2012).

Despite these advancements, many traditional approaches assume uniform illumination of the entire capillary cross-section and neglect the angular dependence of attenuation – a simplification that becomes problematic in modern configurations and strongly absorbing samples. Pauw emphasized that scalar primary-beam transmission corrections may not adequately represent these effects in curved sample holders such as capillaries (Pauw, 2013) and later proposed a modular correction framework to sequence and standardize processing steps, including transmission and self-absorption, but still assumes uniform illumination (Pauw *et al.*, 2017). Bendert et al. and Van Hoesen et al. addressed angular-dependent beam attenuation and secondary scattering in spherical and cylindrical samples with an off-axis beam, respectively (Bendert *et al.*, 2013, Van Hoesen *et al.*, 2019). Their 3D models are general in principle: they integrate over the full sample volume, account for both diffraction angle (2θ) and azimuthal orientation (φ), and consider both primary and secondary scattering contributions. However, they assume a uniform beam profile and neglect the effect of capillary walls. A recent comprehensive methodological overview of SAXS and SANS by Jeffries et al. is also relevant here, as it reinforces the importance of standardized, physically grounded correction procedures (Jeffries *et al.*, 2021).

Taken together, these studies show that absorption correction in capillary-based X-ray scattering is strongly geometry-dependent and that container absorption, beam path lengths, and angular dependence can all be important. However, the existing approaches do not directly cover the specific combination required for the present SWAXS problem. This combination includes a line-collimated Kratky-type geometry, a vertically constrained illuminated volume, experimentally measured non-uniform primary-beam profiles, and explicit treatment of the quartz capillary wall. The present work therefore builds most closely on the path-length integration concept of Bowden and Ryan but adapts it to the actual SWAXS geometry and data-reduction workflow considered here.

Appendix 6 – Experimental and Computational Methods

6.1 Materials

Nonafluorotert-butanol (≥ 99.0 %, Sigma-Aldrich), deionized water and a standard quartz-glass Mark tube with an outer diameter of 1.0 mm and a wall thickness of 0.01 mm (Hilgenberg GmbH; article no. 4017510) were used in the SWAXS measurements. Details on the materials used for the Brij 35/alcohol/water systems discussed in Section 10.3 of Appendix 10 were taken from our previous study

(Cerar et al., 2021); the corresponding densities and attenuation coefficients used in the present reprocessing of these data are listed in Table S1 in Appendix 7.

6.2 SWAXS Measurements

SWAXS profiles were measured using an in-lab-modified Kratky camera (Anton Paar KG, Graz, Austria) connected to a conventional X-ray generator (GE Inspection Technologies, SEIFERT ISO-DEBYEFLEX 3003). In terms of performance, the camera is functionally equivalent to the commercial SAXSess camera (Anton Paar KG, Graz, Austria). The generator operated at 40 kV and 50 mA and was equipped with a Göbel mirror to produce a focused, nominal Cu K_α beam (λ = 0.154 nm). Samples were maintained at 25 °C inside a standard quartz-glass Mark-tube capillary with an outer diameter of 1 mm and a wall thickness of 10 μm. Scattering patterns were recorded using a Fuji BAS 1800II 2D imaging plate system, which offers a spatial resolution of 50 × 50 μm² per pixel and was positioned 267 mm from the central capillary axis; the camera was operated under vacuum with a residual pressure of 2–4 mbar during the measurements and the same vacuum conditions were present inside the capillary during the empty-capillary measurement. Each measurement had a duration of 30 minutes; the imaging plate was read out after a fixed delay of 5 minutes following X-ray exposure. The recorded data were converted to the q-scale, covering a scattering-vector range of 0.01 $Å^{-1} < q < 3.0$ $Å^{-1}$.

Due to the line-collimated geometry of the Kratky-type camera, the experimental SWAXS data are intrinsically affected by instrumental smearing (Glatter, 2018), which remains present even after standard background subtraction and absolute scaling. No numerical desmearing was applied to the experimental data in this study, as this would require solving an ill-posed inverse problem and would introduce additional regularization assumptions. Instead, all theoretical SWAXS curves were numerically subjected to the corresponding instrumental smearing prior to comparison, which represents a well-defined forward procedure based on the experimentally determined primary-beam profiles.

6.3 Molecular Dynamics Simulations and Scattering Intensity Calculations

MD simulations of pure liquid NFTB and water were performed. OPLS-AA (Jorgensen *et al.*, 1996, Watkins & Jorgensen, 2001, Dodda *et al.*, 2017) force-field parameters were used to model NFTB, while the TIP4P (Jorgensen *et al.*, 1983) TIP4P/2005 (Abascal & Vega, 2005), TIP5P (Mahoney & Jorgensen, 2000) and SPC/E (Berendsen *et al.*, 1987) water models were used to model water. The initial configurations contained 4320 NFTB and 33325 water molecules in cubic boxes (side length 10 nm). MD simulations were performed using GROMACS (Abraham *et al.*, 2024). A leap-frog integrator with a 1 fs time step was used and bond lengths involving H-atoms were constrained with LINCS. The temperature was set to 298.15 K using the Nosé-Hoover thermostat and the pressure maintained at 1 bar with the Parrinello-Rahman barostat with time constants of 1 ps and 2 ps respectively. After a steepest descent energy minimization, *NVT* and *NPT* equilibrations were performed, followed by a 5 ns *NPT* production run.

During production, 500 configurations (every 10 ps) were saved for theoretical SWAXS calculations with the complemented system approach (Lajovic *et al.*, 2010). Further details on the MD simulation methodology are available in our previous work (Cerar *et al.*, 2021, Cerar *et al.*, 2017, Tomšič, Jamnik, *et al.*, 2007, Tomšič, Vlcek, *et al.*, 2007, Cerar *et al.*, 2020, Cerar *et al.*, 2019, Tomšič *et al.*, 2019, Tomšič *et al.*, 2018, Lajovic *et al.*, 2010). Before comparison with experimental data, the

calculated theoretical SWAXS curves were subjected to the instrumental smearing procedure described in Section 6.2.

6.4 Computation of Angle-Dependent Transmission Correction Factors

A Python program (https://github.com/jurekovac340/SWAXS-absorption-correction) was developed to calculate the angle-dependent transmission correction factors for SWAXS measurements in horizontal line-collimated geometry presented in Figure 1 and Figure 2 of the main article. The code evaluates equation (11) of the main article, using the weighting and path-length definitions in equations (8)-(21), to account for X-ray attenuation in both the sample and the capillary wall over the constrained illuminated volume. Experimentally determined incident-beam profiles are required and enter the calculation through the weighting function defined in equations (8)-(10); the profiles used here are shown in Figure 3 of the main article. A homogeneous primary beam can also be assumed if desired.

For efficient numerical implementation, the integration domain in equation (11) of the main article was transformed to normalized coordinates on the interval [0,1], yielding an equivalent three-dimensional integral over a unit cube. This enabled the use of the *torchquad* library, which supports high-performance multidimensional integration with GPU acceleration via PyTorch. The integration was performed using the Simpson scheme implemented in *torchquad*, operating on a uniform Cartesian grid with evenly spaced points in all three dimensions. The number of grid points per dimension, n, was chosen so that the total number of evaluation points is $N = n^3$, with $N = 2^{27}$ providing converged results for the absorption correction while remaining computationally tractable; the convergence of the numerical integration is discussed in Section 8.2 of Appendix 8.

The computed transmission values were tabulated for a predefined set of scattering angles. To enable smooth and efficient correction of the experimental scattering profiles, the discrete $A(2\theta)$ values were fitted to a cubic spline, allowing interpolation at arbitrary scattering angles. The absorption correction of the raw experimental data was performed using equation (5) of the main article for each data point in the experimental scattering curve, prior to any further data reduction (e.g., background subtraction). For the absorption correction of sample and water measurements, only scattering events occurring within the actual sample and water scattering volume were considered. This was recognized as a necessary and reasonable simplification, because the liquid-filled capillaries show substantially stronger scattering than the empty capillary over the relevant q-range, as illustrated in Figure S6a. In contrast, for the empty-capillary correction – required for the determination of the scaling factor used to place the SWAXS data on an absolute scale (Orthaber *et al.*, 2000) – only scattering contributions originating from the capillary wall were included. The experimental geometry parameters used in all calculations for our setup were: d = 267 mm, R = 0.49 mm, and t = 0.01 mm.

Importantly, the spline interpolation scheme does not only ensure smooth correction of the experimental profiles but also provides a practical way to adapt the procedure to different samples. In addition, once an instrument-specific set of transmission values has been tabulated for selected attenuation coefficients, the corresponding correction factors for other attenuation coefficients can be efficiently obtained by interpolation. This avoids the need to repeat the full three-dimensional numerical integration and makes the method directly transferable between samples and particularly convenient for routine use. The interpolation can also be performed using simple interpolation expressions instead of the cubic spline method, as described in Appendix 7.

Appendix 7 – Attenuation Coefficients $A(\mu, 2\theta)$ and Interpolation Tables

The linear attenuation coefficients of compounds required for the absorption correction procedure were calculated according to:

$$\mu = \rho\mu_m \,, \tag{S4}$$

where ρ is the density of the compound and μ_m the mass attenuation coefficient (Prince, 2004):

$$\mu_m = \sum_{\mathrm{i}} w_{\mathrm{i}}\mu_{m,\mathrm{i}} \,, \tag{S5}$$

where w_{i} is the mass fraction of element i in the compound and $\mu_{m,\mathrm{i}}$ the mass attenuation coefficient of that element at the wavelength (λ = 0.154 nm in our case). The linear attenuation coefficients of mixtures were obtained by weighting the mass attenuation coefficients of the individual compounds (calculated) and their mass fractions, taking into account the measured density of each mixture. The mass attenuation coefficients of the elements at the wavelength of 0.154 nm were taken from the *Tables of X-ray Mass Attenuation Coefficients* (Hubbell & Seltzer, 2004). The calculated values, together with the corresponding densities for all compounds and mixtures used, are presented in Table S1.

Interpolation between the numerically integrated values of $A(\mu, 2\theta)$ listed in Table S2 allows estimation of A for arbitrary μ and 2θ without full numerical integration. First, the expression (Dwiggins, 1975):

$$A\,(\mu, 2\theta_{\mathrm{i}}) = \exp\left(\sum_{\mathrm{j}=1}^{5} K_{\mathrm{j}}(2\theta_{\mathrm{i}})\mu^{j}\right), \tag{S6}$$

Table S1 X-ray mass attenuation coefficients (for light of wavelength 0.154 nm) and densities used to obtain the linear attenuation coefficients used in this work. The densities of Brij35 mixtures with alcohols and water were determined at 25 °C using an Anton Paar DMA500 density meter.

Compound/mixture/material	μ_m [cm^2 / g]	ρ [g/cm^3] at 25 °C	μ [mm^{-1}]
water	10.130[a]	0.997[b]	1.010
quartz glass	36.814[a]	2.2[c]	8.099
NFTB	12.995[a]	1.693[d]	2.200
EtOH	6.3194[a]	0.7852[e]	0.4962
BuOH	5.3986[a]	0.8054[e]	0.4348
HxOH	4.9822[a]	0.8151[e]	0.4061
5 wt% Brij35–EtOH[g]	6.3168[a]	0.796918 ± 0.000002[f]	0.5034
5 wt% Brij35–BuOH	5.4415[a]	0.813383 ± 0.000004[f]	0.4426
5 wt% Brij35–HxOH	5.0472[a]	0.82462 ± 0.00002[f]	0.4162
5 wt% Brij35–EtOH–2 wt% H_2O	6.3924[a]	0.80283 ± 0.00002[f]	0.5132
5 wt% Brij35–BuOH–2 wt% H_2O	5.5375[a]	0.82113 ± 0.00001[f]	0.4547
5 wt% Brij35–HxOH–2 wt% H_2O	5.1497[a]	0.82821 ± 0.00002[f]	0.4265

[a]Calculated based on (Hubbell & Seltzer, 2004), [b](Tanaka *et al.*, 2001), [c](Nürnberg *et al.*, 2016), [d](Merck Life Science, 2025),[e] (Rodriguez *et al.*, 2001), [f] This work, experimental.
[g]Brij35 (Polyoxyethylene (23) dodecyl ether; $C_{12}E_{23}$; $C_{12}H_{25}$–$(OCH_2CH_2)_{23}$–OH; M ≈ 1198 g/mol); μ_m ≈ 6.27 cm^2 g^{-1}

Table S2 Tabulated $A(\mu, 2\theta)$ values, obtained by numerical integration for our experimental setup with a = 0.1 mm, b = 12 mm and including the experimental primary-beam profiles.

2θ [°]	μ								
	**0.5 mm^{-1}**	**0.75 mm^{-1}**	**1 mm^{-1}**	**1.25 mm^{-1}**	**1.5 mm^{-1}**	**1.75 mm^{-1}**	**2 mm^{-1}**	**2.25 mm^{-1}**	**2.5 mm^{-1}**
**0**	0.52156	0.40855	0.32003	0.25069	0.19637	0.15383	0.1205	0.09439	0.07394
**5**	0.52162	0.40864	0.32014	0.2508	0.19648	0.15393	0.12059	0.09447	0.07401
**10**	0.52189	0.40903	0.32057	0.25125	0.19692	0.15434	0.12096	0.09481	0.07431
**15**	0.52236	0.4097	0.32134	0.25204	0.19769	0.15506	0.12162	0.0954	0.07483
**20**	0.52304	0.41065	0.32243	0.25316	0.19879	0.1561	0.12258	0.09626	0.0756
**25**	0.52389	0.41187	0.32383	0.25463	0.20024	0.15747	0.12385	0.09742	0.07663
**30**	0.5249	0.41335	0.32555	0.25644	0.20203	0.15919	0.12545	0.09888	0.07795
**35**	0.52605	0.41505	0.32756	0.25858	0.20418	0.16127	0.12741	0.10069	0.07959
**40**	0.52729	0.41696	0.32986	0.26106	0.2067	0.16373	0.12975	0.10287	0.08159
**45**	0.52859	0.41904	0.3324	0.26385	0.20957	0.16657	0.13248	0.10545	0.08399
**50**	0.52989	0.42123	0.33515	0.26692	0.21279	0.1698	0.13564	0.10847	0.08683
**55**	0.53115	0.42348	0.33807	0.27025	0.21634	0.17343	0.13923	0.11195	0.09015
**60**	0.53228	0.42571	0.34108	0.27377	0.22017	0.17741	0.14324	0.11589	0.09397
**65**	0.53323	0.42785	0.3441	0.27742	0.22422	0.18171	0.14765	0.1203	0.0983
**70**	0.53392	0.42979	0.34702	0.28107	0.2284	0.18623	0.15238	0.12512	0.10312
**75**	0.5343	0.43147	0.34975	0.28463	0.23259	0.19088	0.15733	0.13025	0.10833
**80**	0.53444	0.43287	0.35223	0.28801	0.23668	0.19552	0.16237	0.13557	0.11382
**85**	0.53457	0.43416	0.35456	0.29124	0.24066	0.2001	0.16742	0.14097	0.11946
**90**	0.53515	0.43567	0.357	0.29451	0.24466	0.2047	0.1725	0.14643	0.1252
**95**	0.53642	0.43762	0.35969	0.29793	0.24874	0.20934	0.17762	0.15192	0.13098
**100**	0.53814	0.43978	0.36246	0.30133	0.25274	0.21387	0.1826	0.15726	0.13659
**105**	0.53985	0.4418	0.36499	0.30444	0.2564	0.21804	0.18719	0.1622	0.1418
**110**	0.54129	0.44346	0.3671	0.30709	0.2596	0.22172	0.19129	0.16663	0.1465
**115**	0.54235	0.4447	0.36877	0.30928	0.26231	0.22491	0.19488	0.17055	0.15067
**120**	0.54308	0.44557	0.37005	0.31107	0.2646	0.22766	0.19801	0.17399	0.15434
**125**	0.54351	0.44614	0.37101	0.31252	0.26653	0.23001	0.20073	0.177	0.15758
**130**	0.54372	0.44647	0.37172	0.31368	0.26815	0.23204	0.20309	0.17963	0.16041
**135**	0.54375	0.44662	0.37223	0.31462	0.26951	0.23377	0.20513	0.18192	0.16289
**140**	0.54366	0.44665	0.37259	0.31538	0.27065	0.23525	0.2069	0.1839	0.16504
**145**	0.54349	0.44659	0.37283	0.31599	0.27161	0.23651	0.2084	0.1856	0.16688
**150**	0.54328	0.44649	0.373	0.31647	0.2724	0.23757	0.20968	0.18704	0.16845
**155**	0.54307	0.44637	0.37312	0.31686	0.27305	0.23845	0.21074	0.18824	0.16975
**160**	0.54287	0.44625	0.3732	0.31717	0.27358	0.23916	0.21159	0.18922	0.1708
**165**	0.5427	0.44615	0.37326	0.31741	0.27398	0.23971	0.21226	0.18997	0.17162
**170**	0.54259	0.44609	0.37331	0.31759	0.27428	0.24011	0.21274	0.19051	0.17221
**175**	0.54253	0.44606	0.37336	0.31772	0.27448	0.24036	0.21304	0.19085	0.17257
**180**	0.54254	0.44609	0.37341	0.31779	0.27458	0.24048	0.21317	0.19099	0.17272

is fitted at discrete angular values $2\theta_\mathrm{i}$ corresponding to those used in the numerical integration procedure. This provides the coefficients $K_\mathrm{j}(2\theta_\mathrm{i})$, which describe the dependence of A on μ for each scattering angle. This step needs to be performed only once for a given instrumental setup.

For a sample with a known attenuation coefficient μ_S, the corresponding values $A\ (\mu_S, 2\theta_\mathrm{i})$ can then be calculated. Finally, the angular dependence of A is fitted for the sample according to:

$$A\ (\mu_S, 2\theta) = \sum_{j=0}^{M} L_j \cdot \sin^{2j}\left(\frac{2\theta}{2}\right), \qquad \text{(S7)}$$

which enables correction of the data at arbitrary scattering angles. The number of terms M should be determined individually for each experimental setup to ensure sufficient agreement with the values obtained by numerical integration. This parameter mainly depends on the capillary radius and wall thickness. In our case (R = 0.49 mm, t = 0.01 mm), a value of $M = 10$ provided a good fit over the relevant $\mu, 2\theta$ range. The maximum relative deviation between the fitted and numerically integrated values was 0.047 % (μ = 2.5 mm^{-1}, q = 0°). The numerically integrated values are compared with the interpolated ones in Figure S2, showing excellent agreement. Utilizing this interpolation scheme together with equation (7) of the main article, we obtained the values of $A(\mu, q)$ listed in Table S3.

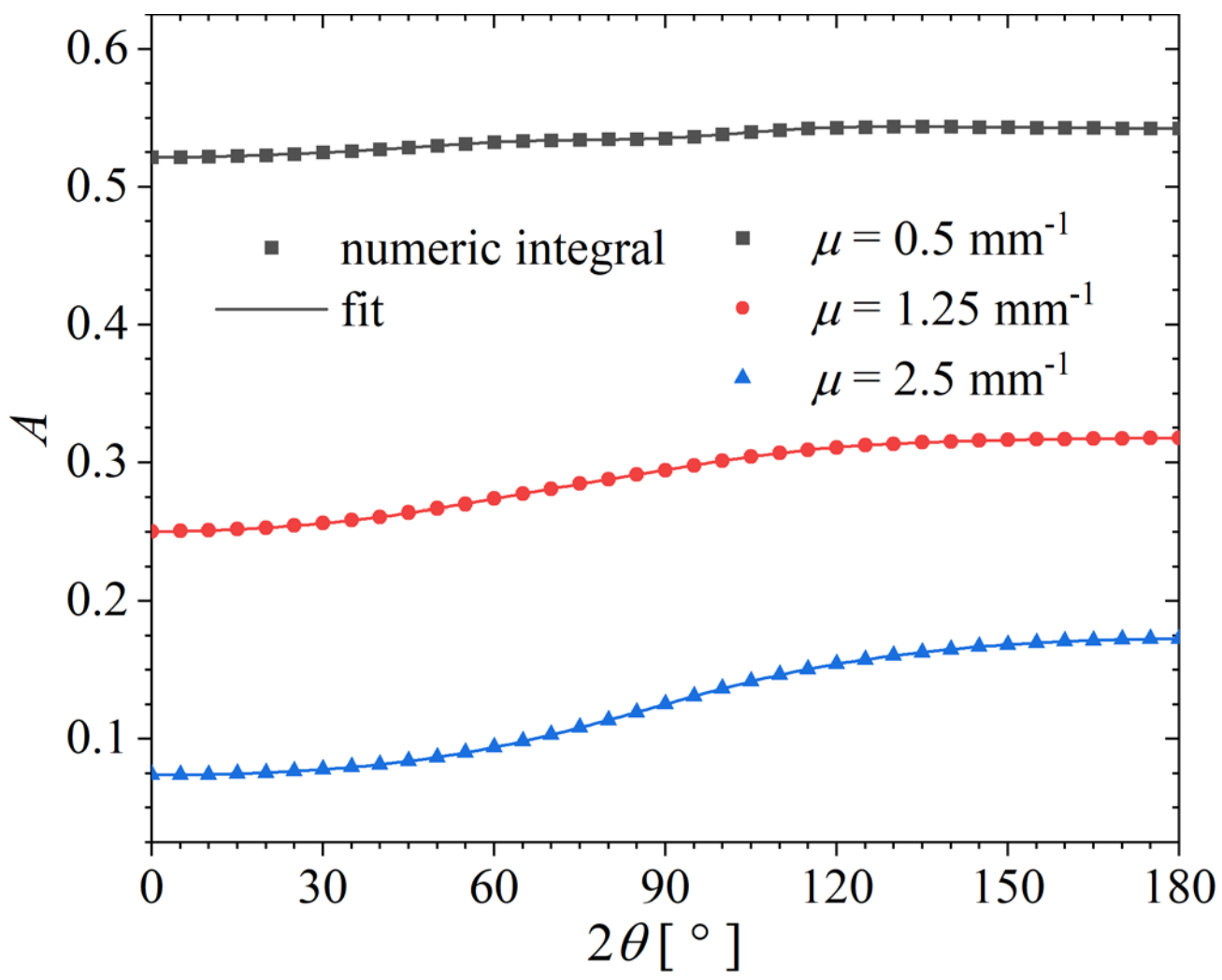

Figure S2. $A(2\theta, \mu)$ values calculated using the numerical integration method (symbols) and the fitted interpolation functions (solid lines) for μ = 0.5, 1.25 and 2.5 mm^{-1}.

Table S3 Tabulated $A(\mu, q)$ values, obtained by numerical integration for our experimental setup with a = 0.1 mm, b = 12 mm and including the experimental primary-beam profiles.

q [nm^{-1}]	μ								
	0.5 mm^{-1}	0.75 mm^{-1}	1 mm^{-1}	1.25 mm^{-1}	1.5 mm^{-1}	1.75 mm^{-1}	2 mm^{-1}	2.25 mm^{-1}	2.5 mm^{-1}
**0**	0.52156	0.40855	0.32003	0.25069	0.19637	0.15383	0.12050	0.09439	0.07394
**4**	0.52167	0.40870	0.32020	0.25086	0.19654	0.15398	0.12064	0.09452	0.07405
**8**	0.52197	0.40915	0.32072	0.25141	0.19708	0.15449	0.12110	0.09493	0.07442
**12**	0.52255	0.40999	0.32168	0.25240	0.19804	0.15539	0.12193	0.09568	0.07508
**16**	0.52344	0.41124	0.32310	0.25387	0.19949	0.15676	0.12319	0.09682	0.07609
**20**	0.52460	0.41287	0.32498	0.25584	0.20143	0.15861	0.12491	0.09838	0.07750
**24**	0.52591	0.41481	0.32727	0.25826	0.20385	0.16095	0.12710	0.10040	0.07933
**28**	0.52732	0.41702	0.32993	0.26113	0.20677	0.16380	0.12982	0.10294	0.08166
**32**	0.52883	0.41949	0.33298	0.26451	0.21026	0.16727	0.13316	0.10610	0.08460
**36**	0.53045	0.42225	0.33648	0.26843	0.21439	0.17144	0.13726	0.11003	0.08831
**40**	0.53206	0.42519	0.34033	0.27288	0.21918	0.17637	0.14218	0.11484	0.09294
**44**	0.53335	0.42803	0.34432	0.27766	0.22449	0.18198	0.14793	0.12058	0.09858
**48**	0.53407	0.43048	0.34815	0.28254	0.23012	0.18813	0.15439	0.12720	0.10522
**52**	0.53431	0.43255	0.35175	0.28738	0.23595	0.19469	0.16148	0.13463	0.11285
**56**	0.53479	0.43470	0.35541	0.29237	0.24203	0.20167	0.16914	0.14281	0.12139
**60**	0.53638	0.43753	0.35955	0.29775	0.24851	0.20907	0.17732	0.15159	0.13062
**64**	0.53918	0.44106	0.36409	0.30336	0.25515	0.21663	0.18564	0.16054	0.14006
**68**	0.54200	0.44426	0.36815	0.30844	0.26124	0.22364	0.19344	0.16897	0.14898
**72**	0.54345	0.44605	0.37083	0.31222	0.26613	0.22952	0.20015	0.17635	0.15688
**76**	0.54368	0.44662	0.37239	0.31497	0.27004	0.23447	0.20596	0.18286	0.16391
**80**	0.54296	0.44630	0.37315	0.31700	0.27329	0.23878	0.21114	0.18870	0.17025
**81**	0.54263	0.44611	0.37325	0.31744	0.27404	0.23980	0.21237	0.19010	0.17176
**81.6**	0.54256	0.44609	0.37341	0.31779	0.27457	0.24047	0.21316	0.19098	0.17271

Appendix 8 – Additional Geometrical and Numerical Checks

8.1 Comparable Primary-Beam Height and Width

As mentioned in the main text, reducing the primary-beam half-width to values comparable to the beam half-height has only a negligible effect on the absorption correction. This is illustrated in a = 0.1 mm and b = 0.1 mm in Figure S3a. In the following, we provide a simple geometrical estimate to quantitatively illustrate this behaviour.

Since absorption depends exponentially on the X-ray path length within the absorbing material, the most relevant quantity is the longest possible path length within the capillary with the sample. If even this maximum path length changes only marginally with a considerable change of the beam width profile, all shorter paths necessarily exhibit even smaller deviations, and the overall absorption correction remains practically unaffected.

For scattering from the extreme position ($x = \pm b$) along the capillary axis, the maximum path length $l_{\max}$ within the capillary can be estimated geometrically from the projection of the capillary diameter $2R$ onto the scattering direction. This leads to the relation:

$$\frac{l_{\max}}{2R} = \frac{1}{\cos\left(\operatorname{atan}\left(\frac{b}{d+R}\right)\right)}, \tag{S8}$$

where the parameters have the same meaning as in the main text of the article. As illustrated in Figure S3b, for our experimental geometry this ratio remains very close to unity even for large values of b. For example, increasing b from 0.1 mm to 12 mm changes $l_{\max}/(2R)$ by only about 0.1%. Consequently, even when the primary-beam half-height and half-width become comparable, the maximum X-ray path length within the absorbing material changes only marginally. This is fully consistent with the negligible effect observed in the angle-dependent transmission coefficient shown in Figure S3a.

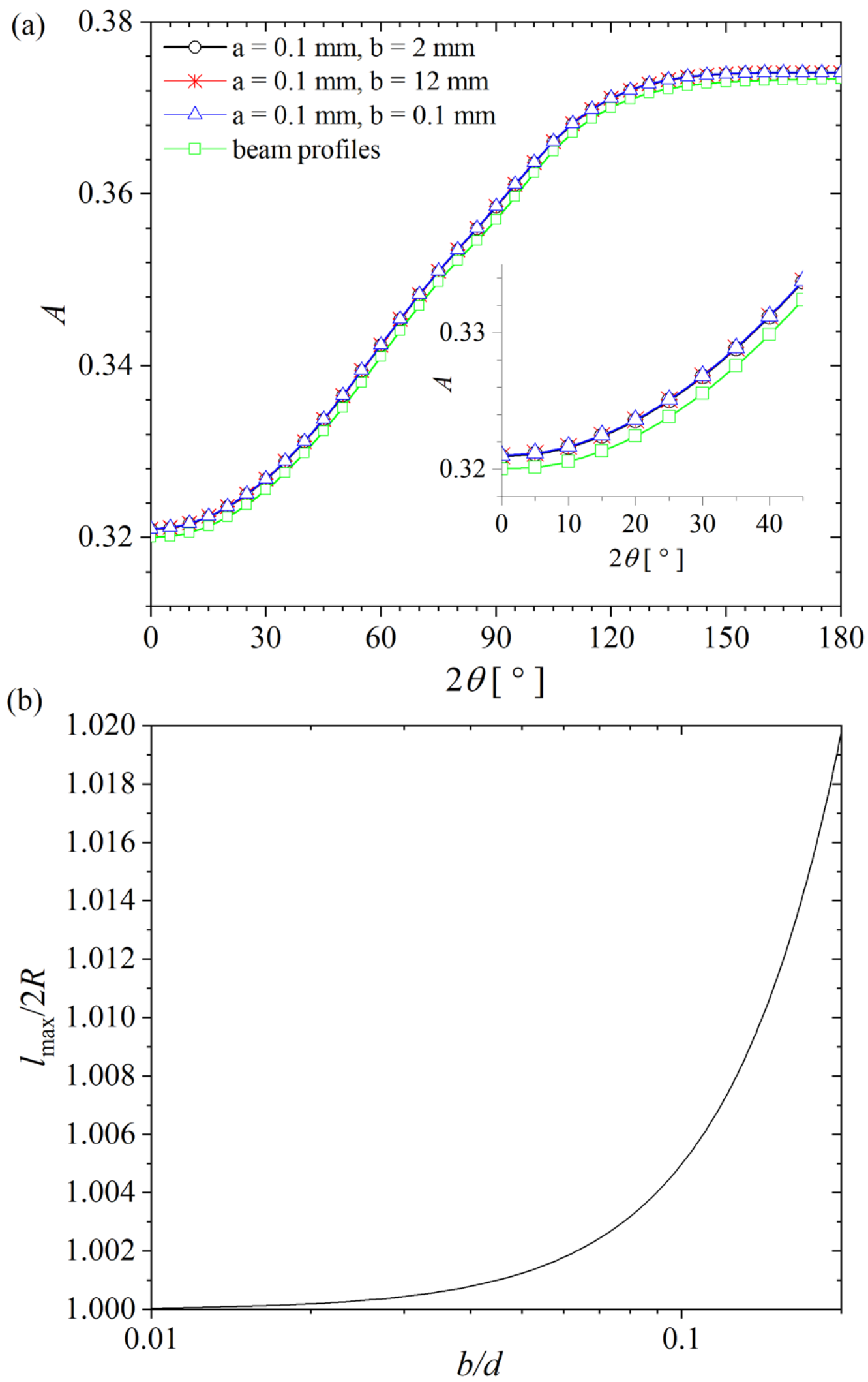

Figure S3. (a) The effect of changing the primary beam half-width b at fixed half-height $a = 0.1$ mm, and comparison with the experimentally measured beam-profile weighting, on the angular dependence of the transmission coefficient $A(2\theta)$, and (b) the ratio of the largest possible path length toward the detector at $2\theta = 0°$ to the capillary diameter as a function of the ratio between the illuminated half-width b and the sample–detector distance d.

8.2 Convergence of the Numerical Integration in Equation (11)

As equation (11) of the main article is evaluated by numerical integration, it is important to check whether the number of evaluation points is sufficient to ensure numerical convergence. The root-mean-square (RMS) error of $A(2\theta)$, calculated from 0 to 180° in 10° steps and expressed as a percentage relative to the result obtained with the highest number of evaluation points ($N = 2^{27}$), is shown in Figure S4. It is plotted as a function of the total number of integration points for several values of the linear attenuation coefficient, ranging from 0.50 mm^{-1} to 5.00 mm^{-1}. The RMS error decreases with increasing N and falls below 0.1 % for all tested values of μ when $N \geq 2^{17}$, indicating high numerical accuracy at this resolution. The error is slightly higher for larger values of μ, with μ = 5.00 mm^{-1} showing the slowest convergence. Nevertheless, even for μ = 5.00 mm^{-1}, the error remains below 0.05% once $N \geq 2^{21}$. These results confirm that the chosen integration approach is numerically stable and justify the sampling density used in the presented absorption-correction procedure.

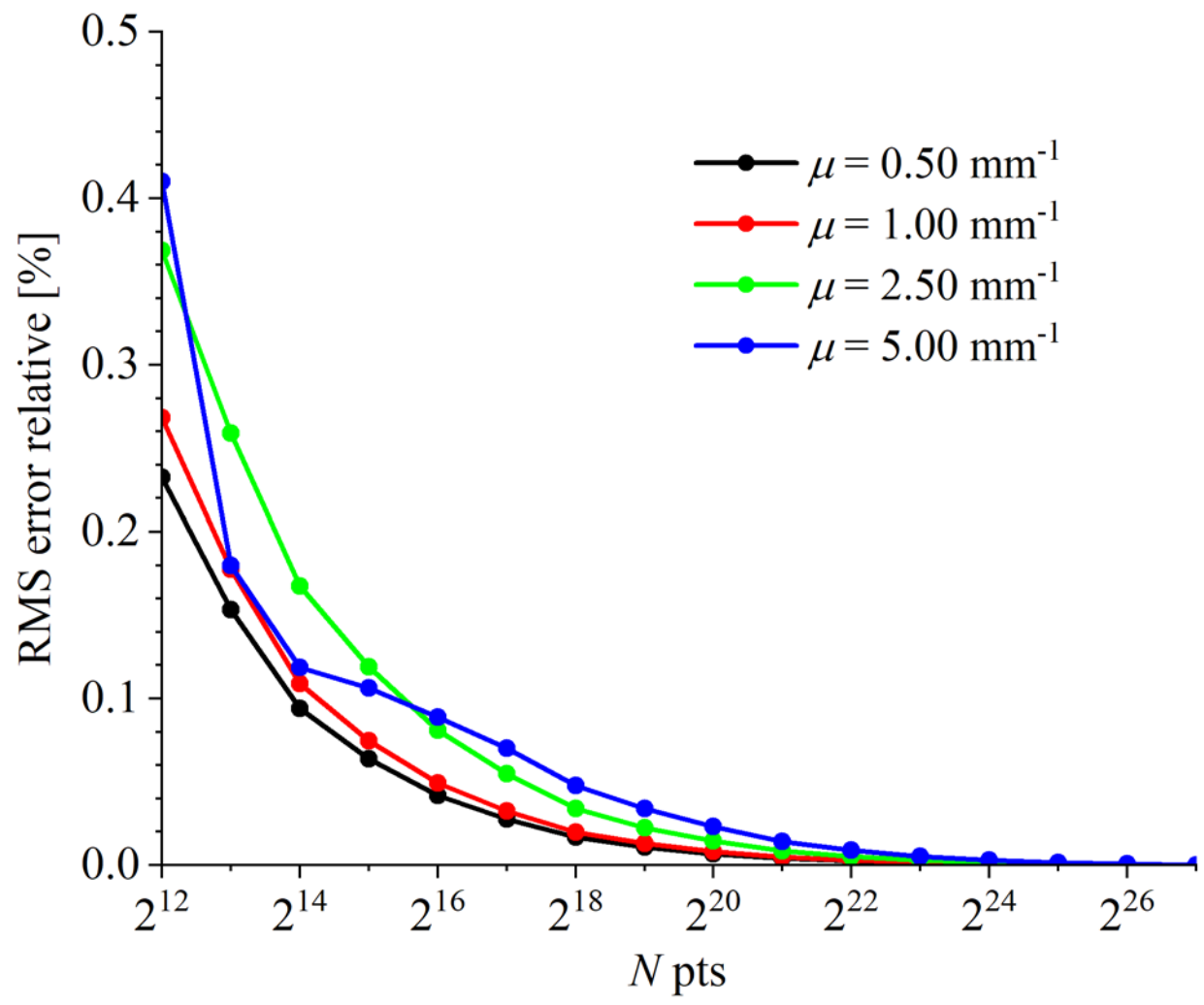

Figure S4. RMS error in $A(2\theta)$, expressed as a percentage, relative to the highest-resolution numerical integration result, as a function of the total number of evaluation points N for different values of the linear attenuation coefficient μ.

8.3 Effect of Capillary Conicity

The influence of capillary conicity on the absorption correction can be assessed by allowing the capillary radius R to vary along the capillary axis x in the model:

$$R(x) = R_0 + \kappa \cdot x \tag{S9}$$

where R_0 is the capillary radius at $x = 0$, and κ is a parameter characterizing the degree of conicity. For simplicity, the capillary wall thickness t is assumed to be constant along the illuminated region.

To quantify the effect of conicity, we consider the maximum relative deviation (relative error) of the transmission coefficient from the cylindrical reference case. We define the relative conicity (expressed in percent) as:

$$\text{conicity} = \left|\frac{R(-b) - R(b)}{R_0}\right| \cdot 100\% \,, \tag{S10}$$

which leads to:

$$\kappa = \frac{\text{conicity}}{100\%} \cdot \frac{R_0}{2b}. \quad \text{(S11)}$$

The geometrical model was modified accordingly to include the axial variation of the capillary radius, and the corresponding transmission coefficients were calculated. The results for an idealized perfectly conical capillary are presented in Figure S5 in terms of the maximum relative error with respect to a cylindrical capillary with $R_0 = 0.49$ mm. The obtained error values are very small, which can be attributed to partial compensation effects: depending on the position along the capillary axis, some paths through the capillary with the sample are shortened (leading to slightly higher transmission), while others are lengthened (leading to slightly lower transmission).

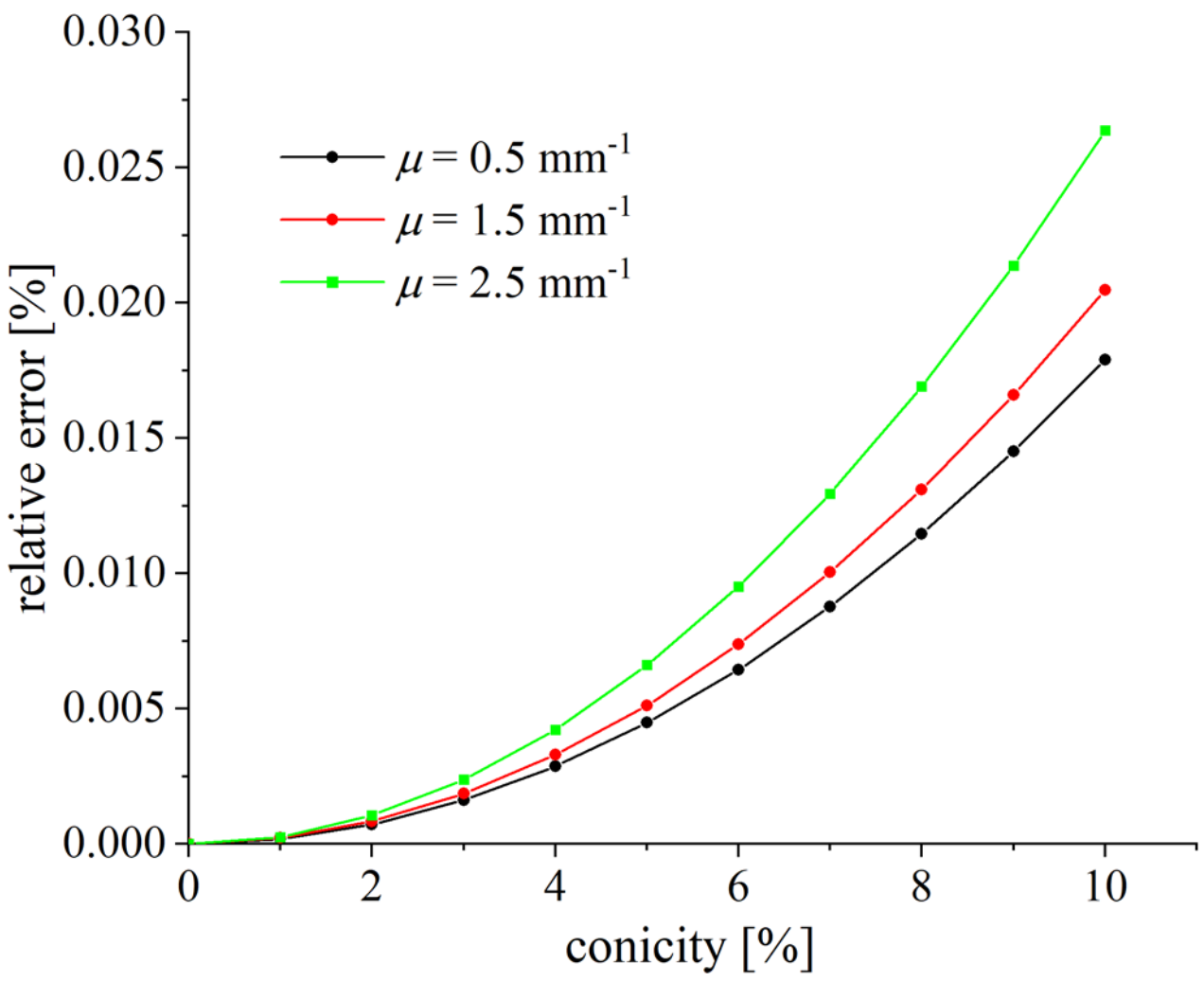

Figure S5. The maximum relative error in the transmission coefficient as a function of conicity at three different values of the linear attenuation coefficient μ.

Appendix 9 – Simplified Beer-Lambert-Type Scalar Transmission Estimate

For comparison purposes, we also consider a deliberately simplified numerical scalar transmission coefficient. It is based on the Beer–Lambert attenuation law and assumes an idealized infinitesimally thin primary beam passing through a sample of uniform thickness defined by the inner diameter of the capillary. In the notation used throughout this work, this simplified transmission factor is given by:

$$A_{\mathrm{BL}} = e^{-\mu_{\mathrm{s}} 2R - \mu_{\mathrm{c}} 2t}, \quad \text{(S12)}$$

where μ_{s} and μ_{c} are the linear attenuation coefficients of the sample and capillary (identical to that used in the full angle-dependent correction), respectively, R is the inner radius of the capillary, such that $2R$ represents the idealized effective sample thickness seen by the thin primary beam, and t is the capillary wall thickness. The coefficient A_{BL} is therefore a scalar, angle-independent numerical transmission factor. Although it is calculated differently from the angle-dependent transmission factor $A(2\theta)$, it is also applied to the raw scattering data using equation (1) of the main article.

This highly simplified treatment was compared with the full angle-dependent $A(2\theta)$ correction in Figure 5 of the main article. Despite its simplicity it yields transmission values very similar to $A(0)$ in the forward-scattering direction and thus helps rationalize why such simplified approaches often remain qualitatively useful in practice. Nevertheless, the deviations from the full $A(2\theta)$ correction become increasingly important at larger scattering angles. The simplified estimate therefore provides a useful qualitative approximation when the angular dependence of the transmission factor is weak, whereas the full angle-dependent correction is required for quantitative SWAXS data reduction.

Appendix 10 –Absorption Correction Examples and Absolute Scaling

This appendix illustrates the practical effects of applying the numerical absorption correction proposed in the present work to the raw SWAXS profiles. It takes up the empty-capillary, water and NFTB datasets introduced in Appendix 4 and focuses on how different correction strategies affect the final reduced data. The water/empty-capillary correction step is then considered separately because it directly affects absolute scaling using water as a secondary standard, before the procedure is also applied to chemically diverse Brij 35/alcohol/water systems as broader experimental examples. The resulting SWAXS curves are compared with the corresponding calculated theoretical SWAXS curves used as references. A simplified Beer-Lambert-based scalar correction, discussed in Appendix 9, is also considered as a qualitatively acceptable correction option.

10.1 Reduced NFTB and Water SWAXS Data

The raw NFTB, water and empty-capillary data shown in Figure S1a in Appendix 4 were corrected using the numerical absorption correction procedure proposed in this study. The resulting corrected SWAXS curves are shown in Figure S6a. These data show that the corrected empty-capillary SWAXS curve lies clearly below the corrected water and NFTB curves over the entire q-range considered. For this correction, the linear attenuation coefficients μ for NFTB, water and quartz were taken as 2.2 mm^{-1}, 1.0 mm^{-1} and 8.1 mm^{-1}, respectively (Hubbell & Seltzer, 2004). A more detailed description of the determination of these attenuation coefficients is given in Appendix 7. With the numerically corrected SWAXS curves, reliable background subtraction (empty-capillary subtraction) and conversion to absolute scale could be performed. The resulting absolute-scale SWAXS data are shown in Figure S6b as open symbols. In comparison to the non-physical NFTB scattering data in Figure S1c and Figure S1d, the data in Figure S6b show a physically reasonable behaviour.

In addition to the reduced experimental SWAXS data on the absolute scale (open circles), Figure S6b also shows the corresponding calculated theoretical SWAXS intensities (solid lines) for pure water and NFTB. These theoretical intensities were obtained using the complemented-system approach described in detail elsewhere (Cerar *et al.*, 2020, Lajovic *et al.*, 2010). Importantly, they are not fitted or adjusted to the experimental data but are directly calculated from molecular configurations obtained from molecular-dynamics simulations. They are used here as an independent theoretical reference to assess whether the reduced experimental SWAXS curves are physically reasonable. In this sense, the comparison in Figure S6b provides a useful consistency check between the numerically reduced experimental data and the independent theoretical model-based curves. Based on our extensive previous

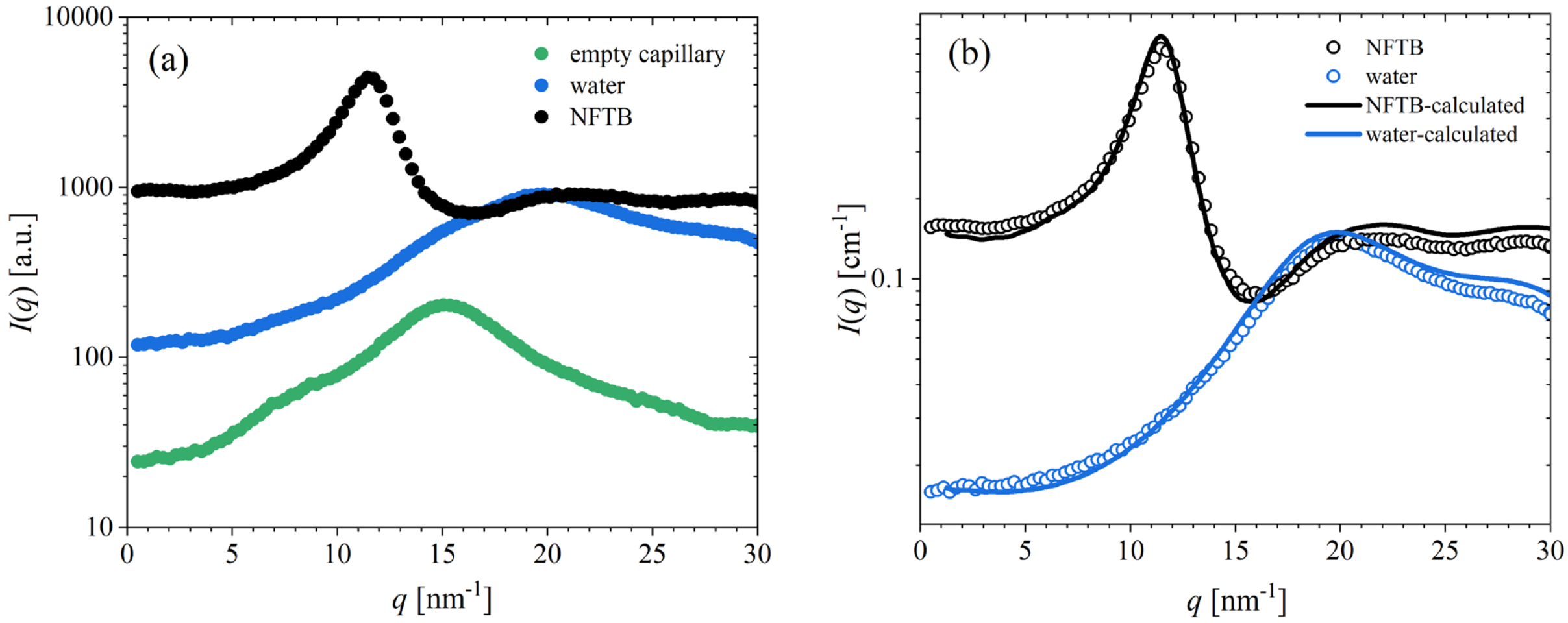

Figure S6. (a) The experimental SWAXS data of NFTB, water and empty capillary after applying the proposed numerical angle-dependent self-absorption correction, and (b) the corrected SWAXS curve of NFTB and water from (a) after capillary subtraction and conversion to absolute scale (open symbols) and the corresponding calculated SWAXS intensities obtained from molecular-dynamics simulations (force field TIP4P/2005 for water and OPLS for NFTB) using the complemented system approach (solid lines).

work on the development and application of the complemented-system approach (Cerar *et al.*, 2021, Cerar *et al.*, 2020, Cerar *et al.*, 2019, Tomšič *et al.*, 2019, Tomšič *et al.*, 2018, Cerar *et al.*, 2017, Lajovic *et al.*, 2012, Lajovic *et al.*, 2009, Tomšič, Jamnik*, et al.*, 2007, Tomšič, Vlcek*, et al.*, 2007), the level of agreement observed between the experimental and theoretical SWAXS intensities in Figure S6b can be regarded as very good. It supports the consistency between the theoretical model-based data and the experimental SWAXS data reduced using the proposed numerical transmission correction procedure. It should also be emphasized that the transmission correction factors $A(2\theta)$ used were calculated solely from the geometry, measured primary-beam profiles and attenuation coefficients, and were not fitted or adjusted to match the theoretical curves. Resolving problems in SWAXS-data reduction of strongly absorbing samples (see Appendix 4 of the SI) was the primary objective of the present study and Figure S6b demonstrates this for NFTB case. Appendix 11 provides an additional independent experimental cross-check of the proposed procedure in the SAXS regime, performed on the same camera and sample but using a more advanced single-photon-counting silicon-strip detector, the Dectris Mythen 1K.

10.2 Exploring Water/Empty-Capillary Scaling Bias

The water dataset is particularly important because it is also used as the secondary standard for absolute scaling. In Figure S6b, only a single theoretical water curve is shown, although we considered several available water models. No single classical water model provides a perfect description of all aspects of liquid water. However, TIP4P/2005 is widely used and offers a balanced description of thermodynamic, structural, and dynamic properties, making it a suitable representative reference in this context. A more detailed comparison of several widely used models is provided in Figure S7.

Not all models reproduce the isothermal compressibility of water equally well, which is important here because the forward-scattering intensity $I(0)$ of liquid water is proportional to the isothermal compressibility. The TIP4P/2005 model performs very well in this respect, as it gives an isothermal compressibility value of 46.3×10^{-6} bar^{-1} at 298 K and 1 bar, close to the experimental value of

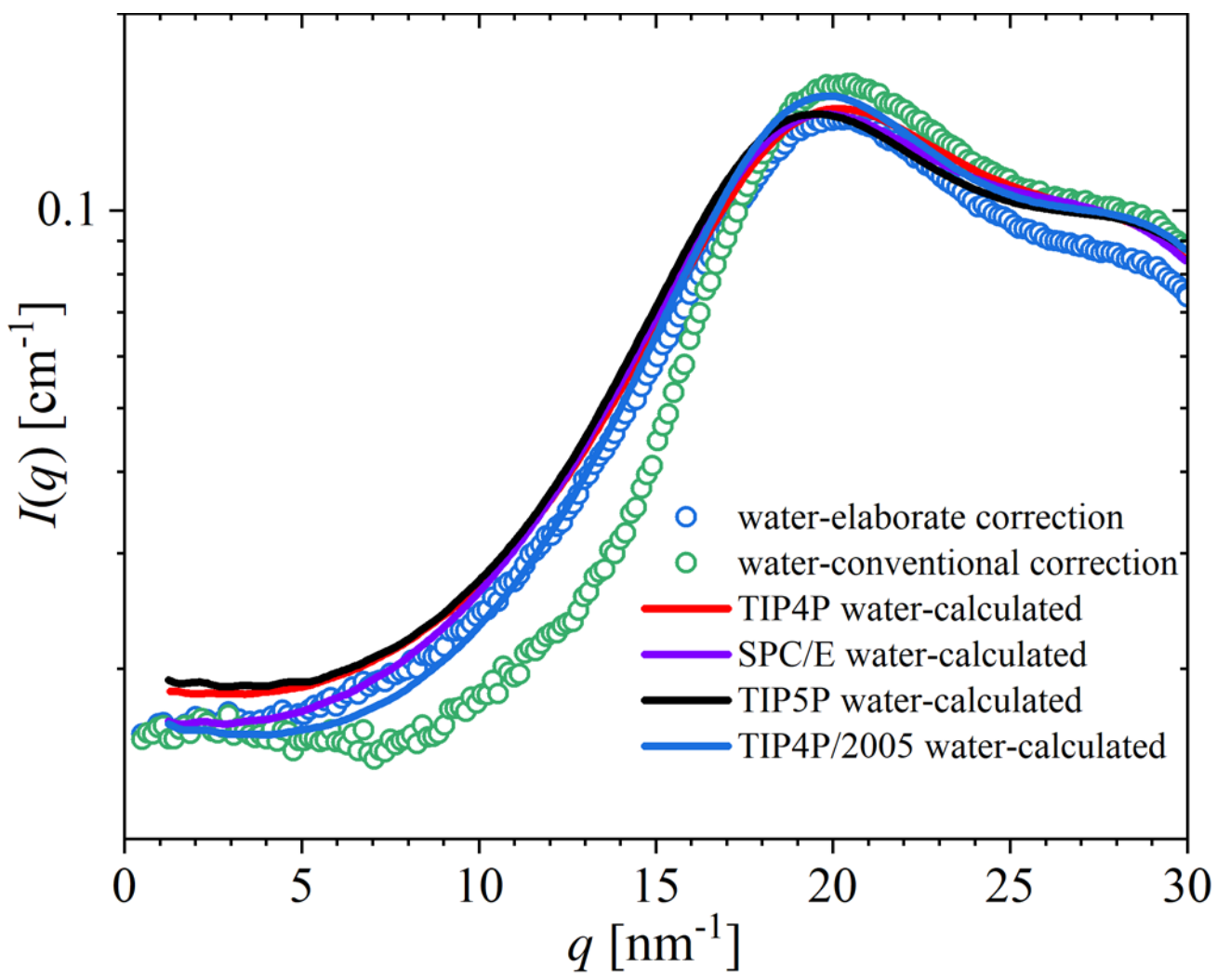

Figure S7. Comparison of final reduced SWAXS curves obtained after background and empty-capillary scattering subtraction following the conventional scalar transmission-correction procedure (green open circles) or the numerical angle-dependent transmission correction proposed in this work (blue open circles), with the theoretical SWAXS intensities calculated for different water force-field models (solid lines) on an absolute scale.

45.3×10^{-6} bar^{-1} (Pi *et al.*, 2009). For the other water models included in the comparison, the reported isothermal compressibility values are 46.1×10^{-6} bar^{-1} for SPC/E, 52.8×10^{-6} bar^{-1} for TIP4P and 57.0×10^{-6} bar^{-1} for TIP5P. Consequently, small differences between the calculated water curves at low-q values Figure S7 mainly reflect model-dependent differences in isothermal compressibility, as expected. Overall, the calculated scattering curves in Figure S7 demonstrate that the general conclusions drawn from Figure S6b are not limited to a specific choice of the water model and therefore further support the model-based consistency check of the proposed procedure.

As follows from equations (3) and (4) of the main article, all experimental data for water in Figure S7 irrespective of the correction type are constrained to the same zero-angle reference value used for absolute-scale calibration ($(d\Sigma/d\Omega)_{w,th}(0) = 1.632 \times 10^{-2}$ cm^{-1} at 293 K) (Orthaber *et al.*, 2000). The relevant difference between the correction procedures observed in Figure S7 is consequently not the absolute intensity level at the lowest q values, but the course of the reduced curve after empty-capillary subtraction at larger q values. In this q range, the scattering curves calculated using different force-field models are very similar and increase smoothly with q.

Interestingly, this behaviour is very similar to that observed for the numerically corrected experimental data (blue open circles) but differs notably from that of the conventionally corrected experimental curve (green open circles). The latter shows a systematic deviation over this part of the q-range, whereas the numerically corrected experimental curve is consistent with the theoretical water curves. This difference can be attributed to the empty-capillary subtraction step. In the conventional workflow, the empty-capillary contribution is scaled using an experimentally measured scalar transmission factor. If this factor is biased, the empty-capillary scattering is not subtracted with the correct weight. In the present case, the direction of the deviation is consistent with over-subtraction of the empty-capillary contribution: the empty-capillary scattering is effectively scaled too strongly relative to the water measurement, which lowers the resulting water curve after background subtraction. Since the water curve is then used to determine the absolute scaling factor, this bias can propagate to all other samples scaled within the same experimental series.

This example shows that even very basic and routinely performed SAXS and SWAXS measurements, such as those of an empty quartz capillary and water, are not immune to the consequences of strongly differing X-ray absorption. According to Figure 5c of the main article, the empty capillary exhibits $A(0) \approx 0.84$, whereas the capillary filled with water exhibits $A(0) \approx 0.32$, corresponding to absorption corrections of approximately 16 and 68 %, respectively. These measurements are crucial for normalizing the data to an absolute scale when water is used as a secondary standard. However, the comparison in Figure S7 shows that conventional scalar transmission correction can lead to systematic deviations even in this routine calibration step when the experimentally determined scalar transmission factors are biased (see Appendix 3).

It is important to emphasize that, because all experimentally corrected SWAXS curves for water are necessarily normalized to the same theoretical $I(0)$ value for water, the critical limitation of the conventional scalar transmission correction becomes apparent only when water is used as a secondary standard for absolute scaling. Namely, for scaling of other samples measured within the same experimental series, for which no independent theoretical reference is available. In such cases, an incorrect transmission-derived scaling factor, resulting from an improper transmission correction of the empty capillary relative to water, propagates directly into a systematic absolute-scaling error of the remaining samples. A clear example of this effect is provided in Figure S8, where the SWAXS curves for alcohols obtained using the conventional scalar correction differ substantially from those scaled using the proposed angle-dependent transmission-correction procedure, despite being measured under otherwise identical experimental conditions. The Beer–Lambert scalar estimate gives a comparable improvement in this respect, as discussed separately in Section 10.4 of Appendix 10.

Within the framework of the present study, these results show that the limitations of conventional scalar transmission correction are not restricted to extreme cases. They can also affect standard reference measurements routinely used for absolute-scale calibration with water as a secondary standard. The comparison presented in Figure S7 therefore underscores the importance of physically consistent absorption correction for reliable background subtraction and absolute-scale SWAXS analysis, even in otherwise routine experimental situations.

10.3 Other Reduced SWAXS Data Examples

For a broader test, the proposed numerical angle-dependent transmission-correction procedure was applied to previously measured SWAXS data for Brij 35/alcohol/water systems from (Cerar *et al.*, 2021). This example study was chosen here because of its versatility, as it applies the same approach to three considerably different systems in terms of structure and complexity, i.e. pure liquid alcohols, a binary surfactant/alcohol system, and a ternary surfactant/alcohol/water system.

Figure S8 compares the originally reported curves, i.e., the theoretical SWAXS intensities and the experimental data corrected using the conventional scalar primary-beam transmission approach, with the experimental curves reprocessed using the proposed numerical angle-dependent transmission correction. The theoretical predictions (solid lines) show a much better agreement with the reprocessed data (dark-coloured symbols) than with the originally reported conventionally reduced data (light-coloured symbols), which were affected by the water/empty-capillary scaling bias discussed in Section 10.2. This improved agreement extends the practical demonstration of the proposed procedure to chemically diverse systems.

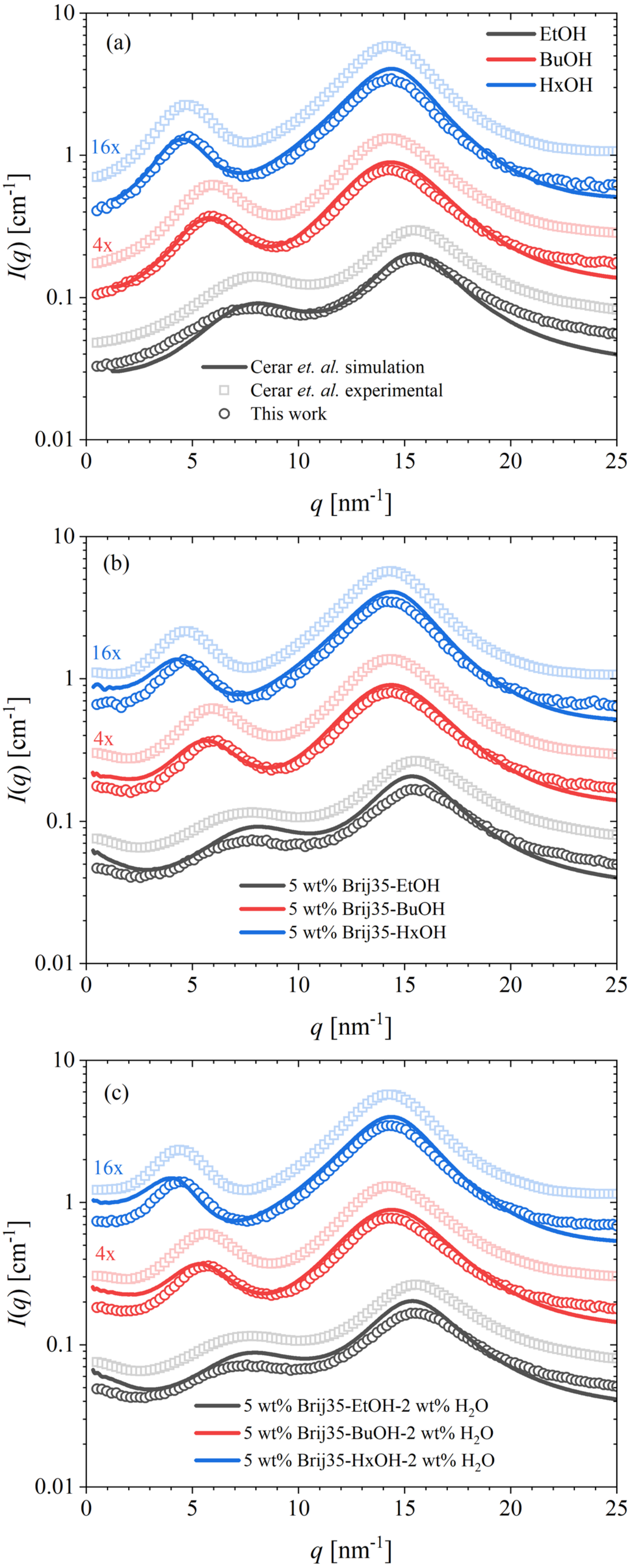

Figure S8. The renormalized experimental SWAXS data (open symbols in full colour) compared to the calculated SWAXS data of our recent study (solid lines) and the originally reported data corrected using the conventional scalar primary-beam transmission approach (open symbols in light colours) (Cerar *et al.*, 2021). For clarity, each group of scattering curves for the same sample was vertically shifted by the indicated factors.

10.4 Simplified Beer-Lambert Estimate Example and Practical Implications

The simplified Beer–Lambert scalar transmission-factor estimate introduced in Appendix 9 and compared with the full $A(2\theta)$ transmission coefficients in Figure 5 of the main article was also applied to the reduction of the water and NFTB SWAXS data. The resulting absolute-scale SWAXS curves are shown in Figure S9 and are compared with those obtained using the full numerical $A(2\theta)$ correction and with the corresponding theoretical SWAXS curves for water and NFTB (TIP4P/2005 water and OPLS-AA for NFTB). The simplified Beer-Lambert estimate gives a similar result in the present q-range, because the angular dependence of $A(2\theta)$ is still modest for this geometry. As indicated by the $A(2\theta)$ transmission coefficients in Figure 5 of the main article, larger deviations are expected at larger scattering angles.

These examples support the practical hierarchy of correction strategies. The conventional experimental scalar primary-beam transmission correction can be sufficient when the measured transmission factors are reliable and when all measurements in a series have similar attenuation. A simplified Beer–Lambert-based scalar estimate can provide a useful approximate correction when the angular dependence of $A(2\theta)$ is weak. For quantitative SWAXS data reduction in cases with substantial absorption differences between sample, solvent, water and empty-capillary measurements, the full numerical $A(2\theta)$ correction is required.

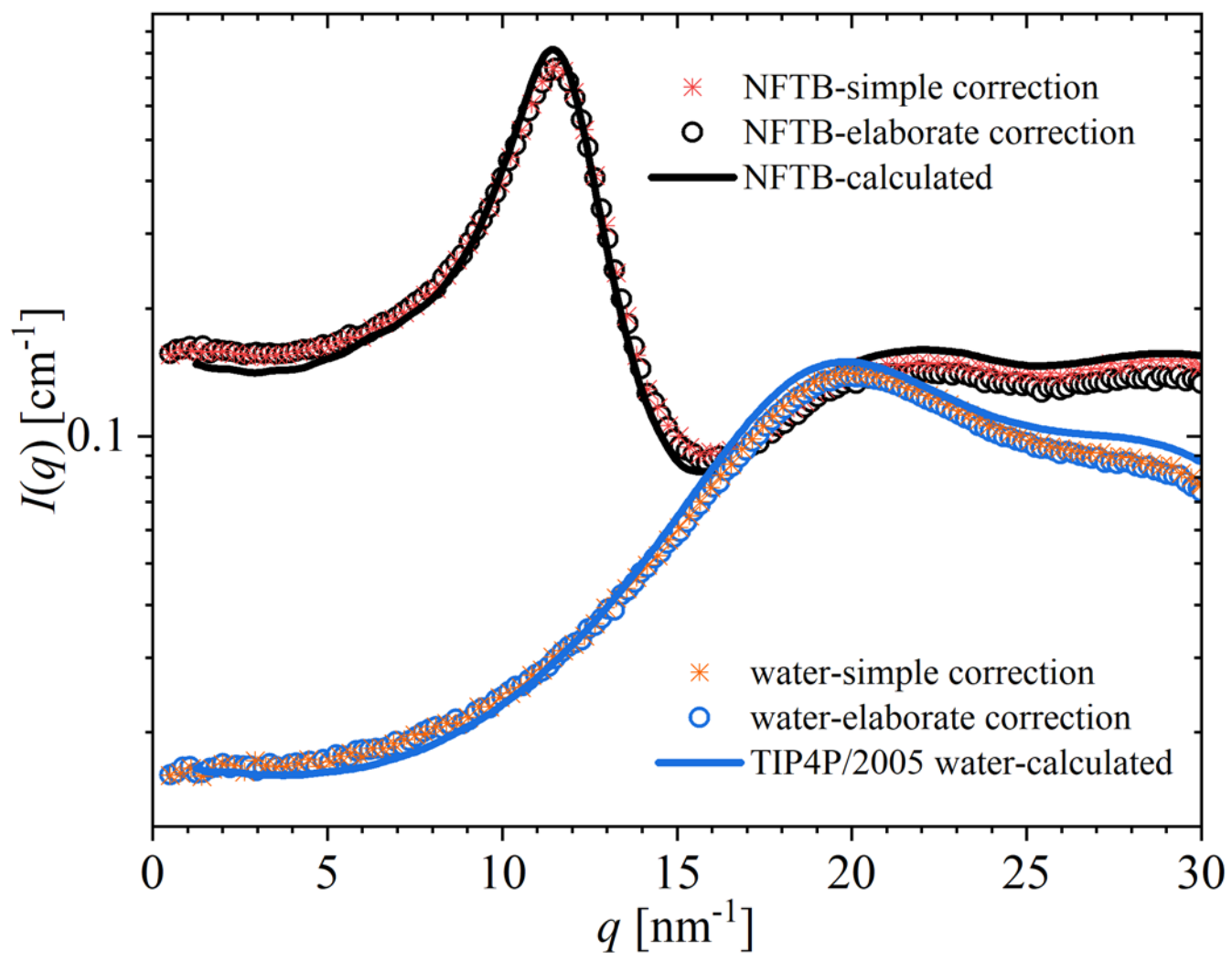

Figure S9. Comparison of different transmission corrections considered in this study on final reduced SWAXS curves (corrected for background and empty capillary scattering) on an absolute scale for water and NFTB using the A_{BL} (simple correction), $A(2\theta)$ (elaborate correction), and calculated theoretical SWAXS data (full lines).

Appendix 11 – Experimental Mythen 1K Detector Cross-Check

As an additional experimental consistency check, the data-reduction workflow was tested using measurements performed with a Dectris Mythen 1K silicon-strip detector on the same SWAXS camera. This check is particularly useful because, in this specific case, it offers a comparison under the same experimental and geometrical conditions but with different detectors, thereby representing two distinct detector-based experiments that remain directly comparable. It is important to understand that if the

experiment were repeated on a different SAXS/SWAXS instrument, the results could not be compared directly. At minimum, the instrumental smearing effects, which are inherent to line-collimated Kratky-type systems, would be different. Therefore, we take the comparison of both experimental results with the independently calculated theoretical scattering intensities with the corresponding instrumental smearing effects included as a direct independent consistency check of the proposed absorption-correction procedure.

The results are shown in Figure S10 for water and NFTB. The Fujifilm BAS 1800 II imaging-plate data cover the full SAXS/SWAXS range shown, whereas the Mythen 1K data span only the accessible SAXS region. These results show that the conventional transmission-based correction remains sensitive to radiation-hardening-related biases for both detector systems used. This is most clearly seen for NFTB in Figure S10b, where the conventionally corrected imaging-plate data (black open circles) and Mythen 1K data (blue open circles) are both notably inconsistent with the independently calculated theoretical SWAXS intensities on an absolute scale (grey curve). The

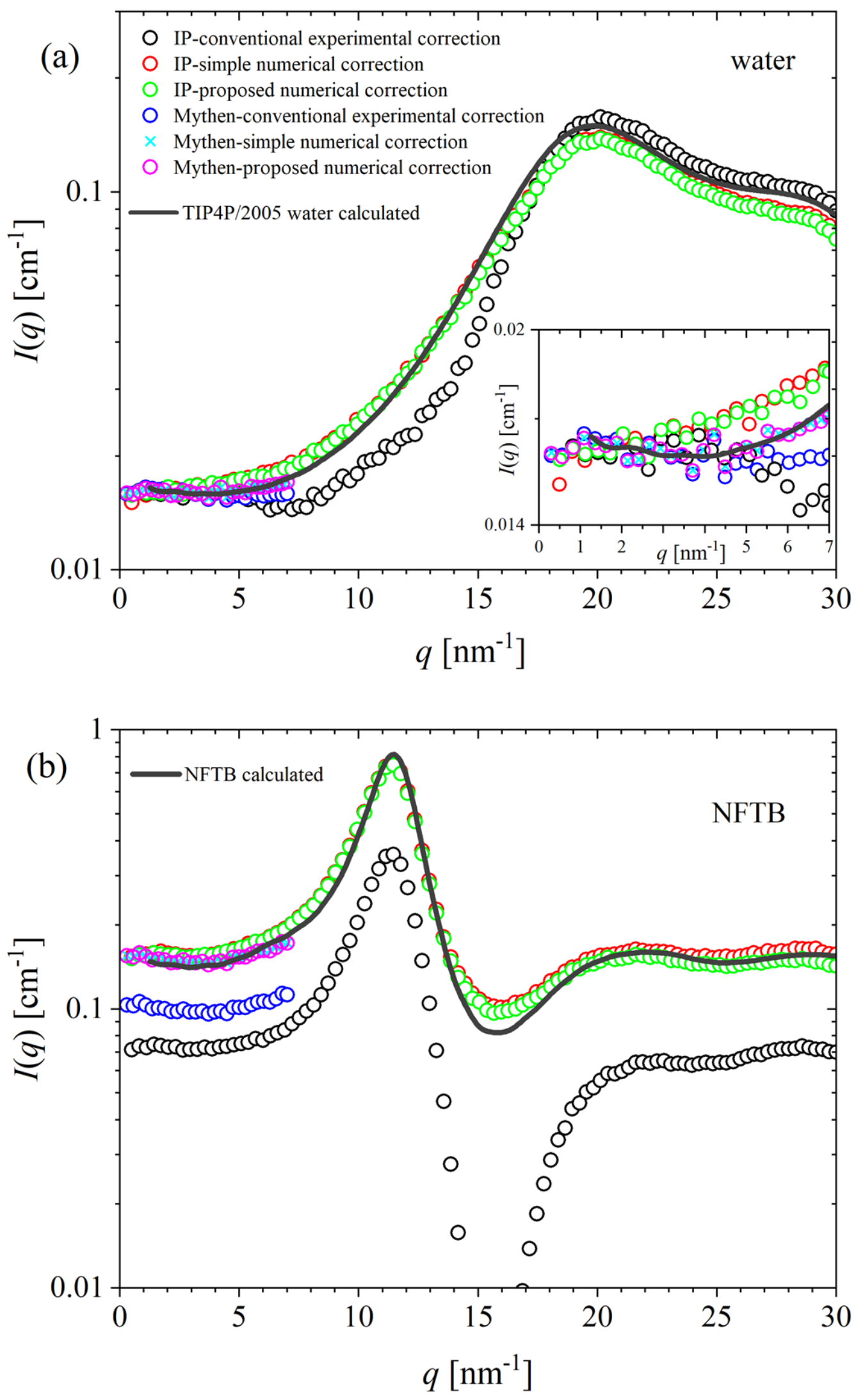

Figure S10. Comparison of data reduction and absolute scaling obtained with the Fujifilm BAS 1800 II imaging-plate detector and the Dectris Mythen 1K silicon-strip detector on the same Kratky-type SWAXS camera and with the same sample/capillary arrangement. The imaging-plate data cover the full SAXS/SWAXS range shown, while the Mythen 1K data provide an independent cross-check in the accessible SAXS region. Results are shown for (a) water and (b) NFTB.

somewhat larger deviation of the imaging-plate data from the theoretical grey curve than for the corresponding Mythen 1K data indicates that the latter detector system is affected to a somewhat smaller extent. This is consistent with the fact that the Mythen 1K detector can partly suppress the contribution of harder X-rays, but not completely.

Importantly, this observation should not be interpreted as a limitation specific to the Kratky-type SWAXS camera geometry used here. Rather, it reflects a more general limitation of transmission measurements when a spectrally non-ideal primary beam is strongly attenuated and then recorded by a detector with finite energy discrimination. As discussed in Appendix 3, radiation-hardening effects and related harmonic-induced biases in SAXS transmission measurements with semi-transparent beamstops have recently been addressed even for synchrotron SAXS instrumentation (Wu & Li, 2024).

In our case, the same radiation-hardening related spectral modification occurs in our SWAXS instrument for both detectors but is registered differently by the two detectors. This leads to detector-dependent biases in the data, which affect both the conventional experimental scalar transmission correction and the subsequent absolute scaling using water as a secondary standard, as evident from the data in Figure S10b. In contrast, applying the proposed numerical absorption correction to the raw NFTB data makes the datasets from both detectors mutually consistent (green and magenta open circles in Figure S10b). The corrected datasets also practically overlap with the theoretical SWAXS data (grey curve in Figure S10b). This supports the conclusion that for a strongly absorbing sample such as NFTB, where the conventional scalar primary-beam transmission correction clearly fails (see Figure S1d in Appendix 4), the proposed numerical correction provides a consistent data-reduction result.

For the water data in Figure S10a, these effects are not evident from the absolute intensity levels, because water is used as the secondary standard for absolute-scale calibration and is therefore scaled to the same theoretical value irrespective of the detector system. However, slight differences can still be noticed in the shape of the processed curves (e.g., the conventionally corrected black and blue open circles in comparison to the numerically corrected green and magenta open circles in Figure S10a). These shape differences indicate that the empty-capillary contribution was subtracted with a different effective weight during background subtraction of the water data. In other words, in the conventionally corrected data, the empty-capillary contribution is overestimated, and these curves therefore lie noticeably below the theoretical black curve over the central part of the q-range in Figure S10a.

In Figure S10a, the red open circles represent the data corrected according to the simplified Beer-Lambert-based scalar correction procedure. Compared with the green open circles, which represent the full angle-dependent numerically corrected data, only a minor difference is observed between the curves. This indicates that, in the measured q-range, corresponding to a scattering angle up to approximately 43 °, the angular dependence of the absorption correction is small in this case.

We can conclude that for NFTB and water, the Mythen 1K data corrected using the numerical absorption-correction procedure are consistent with the corresponding imaging-plate data and with the calculated theoretical SWAXS intensities. These results therefore indicate that the data-reduction workflow based on calculated transmission factors also works in cases where the conventional experimental correction procedure clearly fails. In addition, the detector-substitution experiment provides an additional consistency check between the transmission correction, empty-capillary subtraction and absolute-scale normalization.

The correction factors used in this comparison were not fitted, tuned or adjusted to match either the Mythen 1K data or the independently calculated theoretical curves. They were determined solely from the experimental geometry, measured primary-beam profiles and attenuation coefficients used in the numerical procedure. The Mythen 1K measurement therefore provides an additional direct cross-check of the proposed correction approach.

References

Abascal, J. L. & Vega, C. (2005). *J. Chem. Phys.* **123**.

Abraham, M., Alekseenko, A., Bergh, C., Blau, C., Briand, E., Doijade, M., Fleischmann, S., Gapsys, V., Garg, G., Gorelov, S., Gouaillardet, G., Gray, A., Irrgang, M. E., Jalalypour, F., Jordan, J., Junghans, C., Kanduri, P., Keller, S., Kutzner, C., Lemkul, J. A., Lundborg, M., Merz, P., Miletić, V., Morozov, D., Páll, S., Schulz, R., Shirts, M., Shvetsov, A., Soproni, B., van der Spoel, D., Turner, P., Uphoff, C., Villa, A., Wingbermühle, S., Zhmurov, A., Bauer, P., Hess, B. & Lindahl, E. (2024). *Zenodo*.

Baur, M., Uhlmann, N., Pöschel, T. & Schröter, M. (2019). *Rev. Sci. Instrum.* **90**.

Bendert, J., Blodgett, M. & Kelton, K. (2013). *Found. Crystallogr.* **69**, 131-139.

Berendsen, H. J., Grigera, J.-R. & Straatsma, T. P. (1987). *Journal of Physical Chemistry* **91**, 6269-6271.

Bergmann, A., Orthaber, D., Scherf, G. & Glatter, O. (2000). *J. Appl. Crystallogr.* **33**, 869-875.

Bond, W. (1959). *Acta Crystallogr.* **12**, 375-381.

Bowden, M. & Ryan, M. (2010). *J. Appl. Crystallogr.* **43**, 693-698.

Bradley, A. (1935). *Proc. Phys. Soc.* **47**, 879.

Brooks, R. A. & Di Chiro, G. (1976). *Physics in medicine & biology* **21**, 390-398.

Cerar, J., Jamnik, A., Pethes, I., Temleitner, L., Pusztai, L. & Tomšič, M. (2020). *J. Colloid Interface Sci.* **560**, 730-742.

Cerar, J., Jamnik, A., Szilágyi, I. & Tomšič, M. (2021). *J. Colloid Interface Sci.* **594**, 150-159.

Cerar, J., Jamnik, A. & Tomšič, M. (2019). *J. Mol. Liq.* **276**, 307-317.

Cerar, J., Lajovic, A., Jamnik, A. & Tomšič, M. (2017). *J. Mol. Liq.* **229**, 346-357.

Chen, Y., Wei, L., Qian, F., Yang, Z., Wang, S., Wu, Y., Zhang, Q., Fan, Q. & Cao, L. (2018). *Chinese Physics B* **27**, 024101.

Claassen, A. (1930). *Philos. Mag.* **9**, 57-65.

Dodda, L. S., Vilseck, J. Z., Tirado-Rives, J. & Jorgensen, W. L. (2017). *J. Phys. Chem. B.* **121**, 3864-3870.

Dwiggins, C. (1972). *Found. Crystallogr.* **28**, 219-220.

Dwiggins, C. (1975). *Found. Crystallogr.* **31**, 146-148.

Glatter, O. (2018). *Scattering Methods and their Application in Colloid and Interface Science*. Amsterdam: Elsevier.

Hövelmann, J., Stawski, T. M., Besselink, R., Freeman, H. M., Dietmann, K. M., Mayanna, S., Pauw, B. R. & Benning, L. G. (2019). *Nanoscale* **11**, 6939-6951.

Hubbell, J. & Seltzer, S. (2004). *Gaithersburg, MD*.

Jeffries, C. M., Ilavsky, J., Martel, A., Hinrichs, S., Meyer, A., Pedersen, J. S., Sokolova, A. V. & Svergun, D. I. (2021). *Nat. Rev. Methods Primers* **1**, 70.

Jorgensen, W. L., Chandrasekhar, J., Madura, J. D., Impey, R. W. & Klein, M. L. (1983). *J. Chem. Phys.* **79**, 926-935.

Jorgensen, W. L., Maxwell, D. S. & Tirado-Rives, J. (1996). *J. Am. Chem. Soc.* **118**, 11225-11236.

Kirschbaum, K., Martin, A. & Pinkerton, A. A. (1997). *J. Appl. Crystallogr.* **30**, 514-516.

Lajovic, A., Tomsic, M., Fritz-Popovski, G., Vlcek, L. & Jamnik, A. (2009). *J. Phys. Chem. B.* **113**, 9429-9435.

Lajovic, A., Tomšič, M. & Jamnik, A. (2010). *J. Chem. Phys.* **133**, 174123.

Lajovic, A., Tomšič, M. & Jamnik, A. (2012). *Acta Chim. Slov.* **59**.

Mahoney, M. W. & Jorgensen, W. L. (2000). *J. Chem. Phys.* **112**, 8910-8922.

Merck Life Science, L. (2025). Sigma-Aldrich.

Møller, E. & Jensen, E. (1952). *Acta Crystallogr.* **5**, 345-348.

Nürnberg, F., Kühn, B. & Rollmann, K. (2016). *Laser-Induced Damage in Optical Materials 2016*, pp. 42-54. SPIE.

Oberta, P., Platonov, Y. & Flechsig, U. (2012). *Synchrotron Radiation* **19**, 675-681.

Orthaber, D., Bergmann, A. & Glatter, O. (2000). *J. Appl. Crystallogr.* **33**, 218-225.

Pauw, B. R. (2013). *J. Phys.: Condens. Matter* **25**, 383201.

Pauw, B. R., Smith, A., Snow, T., Terrill, N. & Thünemann, A. (2017). *J. Appl. Crystallogr.* **50**, 1800-1811.

Pi, H. L., Aragones, J. L., Vega, C., Noya, E. G., Abascal, J. L., Gonzalez, M. A. & McBride, C. (2009). *Mol. Phys.* **107**, 365-374.

Polikarpov, M., Snigireva, I. & Snigirev, A. (2014). *Synchrotron Radiation* **21**, 484-487.

Prince, E. (2004). *International Tables for Crystallography, Volume C: Mathematical, physical and chemical tables*. Springer Science & Business Media.

Ritter, H., Harris, R. & Wood, R. (1951). *J. Appl. Phys.* **22**, 169-176.

Rodriguez, A., Canosa, J. & Tojo, J. (2001). *J. Chem. Eng. Data.* **46**, 1506-1515.

Schmitt, D. & Ouladdiaf, B. (1998). *J. Appl. Crystallogr.* **31**, 620-624.

Shimizu, K. & Omote, K. (2008). *Rigaku J* **24**, 1-9.

Sulyanov, S., Gogin, A. & Boysen, H. (2012). *J. Appl. Crystallogr.* **45**, 93-97.

Tanaka, M., Girard, G., Davis, R., Peuto, A. & Bignell, N. (2001). *Metrologia* **38**, 301.

Tomšič, M., Cerar, J. & Jamnik, A. (2018). *J. Mol. Liq.* **259**, 291-303.

Tomšič, M., Cerar, J. & Jamnik, A. (2019). *J. Colloid Interface Sci.* **557**, 328-335.

Tomšič, M., Jamnik, A., Fritz-Popovski, G., Glatter, O. & Vlček, L. (2007). *J. Phys. Chem. B.* **111**, 1738-1751.

Tomšič, M., Vlcek, L., Jamnik, A. & Fritz-Popovski, G. (2007). *Acta Chim. Slov.* **54**, 484-491.

Van de Casteele, E., Van Dyck, D., Sijbers, J. & Raman, E. (2002). *Physics in Medicine & Biology* **47**, 4181-4190.

Van Hoesen, D. C., Bendert, J. C. & Kelton, K. F. (2019). *Found. Crystallogr.* **75**, 362-369.

Watkins, E. K. & Jorgensen, W. L. (2001). *J. Phys. Chem. A.* **105**, 4118-4125.

Wu, H. & Li, Z. (2024). *Synchrotron Radiation* **31**.
